# Guidelines for Biobanking of Bone Marrow Adipose Tissue and Related Cell Types: Report of the Biobanking Working Group of the International Bone Marrow Adiposity Society

**DOI:** 10.3389/fendo.2021.744527

**Published:** 2021-09-27

**Authors:** Stephanie Lucas, Michaela Tencerova, Benoit von der Weid, Thomas Levin Andersen, Camille Attané, Friederike Behler-Janbeck, William P. Cawthorn, Kaisa K. Ivaska, Olaia Naveiras, Izabela Podgorski, Michaela R. Reagan, Bram C. J. van der Eerden

**Affiliations:** ^1^ Marrow Adiposity and Bone Lab-MABLab ULR4490, Univ. Littoral Côte d’Opale, Boulogne-sur-Mer, Univ. Lille, CHU Lille, Lille, France; ^2^ Molecular Physiology of Bone, Institute of Physiology of the Czech Academy of Sciences, Prague, Czechia; ^3^ School of Life Sciences, École Polytechnique Fédérale de Lausanne, Lausanne, Switzerland; ^4^ Swiss Institute of Bioinformatics, Lausanne, Switzerland; ^5^ Department of Biomedical Sciences, Faculty of Biology and Medicine, Université de Lausanne, Lausanne, Switzerland; ^6^ Clinical Cell Biology, Department of Pathology, Odense University Hospital, Odense, Denmark; ^7^ Clinical Cell Biology, Pathology Research Unit, Department of Clinical Research, University of Southern Denmark, Odense, Denmark; ^8^ Department of Molecular Medicine, University of Southern Denmark, Odense, Denmark; ^9^ Department of Forensic Medicine, Aarhus University, Aarhus, Denmark; ^10^ Institute of Pharmacology and Structural Biology, Université de Toulouse, CNRS UMR 5089, Toulouse, France; ^11^ Equipe labellisée Ligue contre le cancer, Toulouse, France; ^12^ Department of Biochemistry and Molecular Cell Biology, University Medical Center Hamburg-Eppendorf, Hamburg, Germany; ^13^ Department of Orthopedics, University Medical Center Hamburg-Eppendorf, Hamburg, Germany; ^14^ British Heart Foundation Centre for Cardiovascular Science, The Queen’s Medical Research Institute, University of Edinburgh, Edinburgh, United Kingdom; ^15^ Institute of Biomedicine, University of Turku, Turku, Finland; ^16^ Hematology Service, Departments of Oncology and Laboratory Medicine, Lausanne University Hospital (CHUV), Université de Lausanne, Lausanne, Switzerland; ^17^ Department of Pharmacology, Wayne State University School of Medicine and Karmanos Cancer Institute, Detroit, MI, United States; ^18^ Center for Molecular Medicine, Maine Medical Center Research Institute, Scarborough, ME, United States; ^19^ Graduate School for Biomedical Science, Tufts University, Boston, MA, United States; ^20^ Laboratory for Calcium and Bone Metabolism, Department of Internal Medicine, Erasmus University Medical Center, Rotterdam, Netherlands

**Keywords:** bone marrow adiposity, bone marrow adipocytes, bone marrow stromal cells, biobanking, cell isolation protocols, international research networks, patient information, clinical studies

## Abstract

Over the last two decades, increased interest of scientists to study bone marrow adiposity (BMA) in relation to bone and adipose tissue physiology has expanded the number of publications using different sources of bone marrow adipose tissue (BMAT). However, each source of BMAT has its limitations in the number of downstream analyses for which it can be used. Based on this increased scientific demand, the International Bone Marrow Adiposity Society (BMAS) established a Biobanking Working Group to identify the challenges of biobanking for human BMA-related samples and to develop guidelines to advance establishment of biobanks for BMA research. BMA is a young, growing field with increased interest among many diverse scientific communities. These bring new perspectives and important biological questions on how to improve and build an international community with biobank databases that can be used and shared all over the world. However, to create internationally accessible biobanks, several practical and legislative issues must be addressed to create a general ethical protocol used in all institutes, to allow for exchange of biological material internationally. In this position paper, the BMAS Biobanking Working Group describes similarities and differences of patient information (PIF) and consent forms from different institutes and addresses a possibility to create uniform documents for BMA biobanking purposes. Further, based on discussion among Working Group members, we report an overview of the current isolation protocols for human bone marrow adipocytes (BMAds) and bone marrow stromal cells (BMSCs, formerly mesenchymal), highlighting the specific points crucial for effective isolation. Although we remain far from a unified BMAd isolation protocol and PIF, we have summarized all of these important aspects, which are needed to build a BMA biobank. In conclusion, we believe that harmonizing isolation protocols and PIF globally will help to build international collaborations and improve the quality and interpretation of BMA research outcomes.

## 1 Introduction

Over the last two decades there has been an increased interest by researchers to study bone marrow adipose tissue (BMAT) in relation to bone and adipose tissue physiology, which expanded the number of publications in the literature. Researchers have used different sources of BMAT to address the major questions in the bone marrow adiposity (BMA) field including: 1) What is the role of BMAT and how does its function change in different physiological and pathophysiological conditions compared to white or brown adipose tissues? and 2) How do we employ bone marrow adipocytes (BMAds) and bone marrow stromal cells (BMSCs) to enrich our knowledge about BMA? However, the methods used to isolate BMAT, BMAds and BMSCs, and the anatomical site(s) from which these are obtained, may influence their biological properties and the downstream analyses for which they can be used reliably. BMA is a young field that has recently received increasing attention among numerous scientific communities (1–5). The goal of this BMA biobanking position paper is to make protocols accessible for all interested researchers and to support researchers in designing their biobanks, sharing their samples and expertise to accelerate progress in BMA research. By facilitating the exchange of knowledge and biological samples, we aim to enhance BMA research worldwide. The BMA biobanking initiative was formed based on discussions among scientists involved in the International Bone Marrow Adiposity Society (BMAS), who created a BMAS Working Group (WG) focused on a need to coordinate biobanking activities in the field; develop guidelines and steps to advance establishment of biobanks for BMA research; and to enhance transparency about differences in BMA-related protocols used among the growing BMA research community. Our Biobanking WG identified currently used collection procedures related to BMAT, BMAds and BMSCs isolated from bone marrow (BM) aspirates, BM plasma and bone tissues. Since samples might be shared among international collaborators, it is critical to consider aligning procedures as much as possible for all sites participating in BMA research, and where access to samples and data will be subject to the strictest scientific and ethical scrutiny. The WG realizes that this is currently impossible to achieve, but as first attempts towards harmonization for biobanks have been successfully employed to access cross-institutional data (6), we aim to report here on the uniformities and discrepancies between protocols and storage of materials related to BMA. Our effort is in line with the recently published BMAS guidelines on nomenclature, abbreviations and units (7), as well as data reporting guidelines and methodological standards (8), produced by the BMAS working groups on Nomenclature and Methodologies, respectively.

Ethical, legal and social issues are complex, affect many biobanking aspects, and have not been fully resolved by the scientific community. In this review, we will also address guidelines in regard to BMA-related biobanking issues, such as informed consent, and compare differences in biobanking procedures and institutional patient information forms (PIFs).

**Figure 1:**
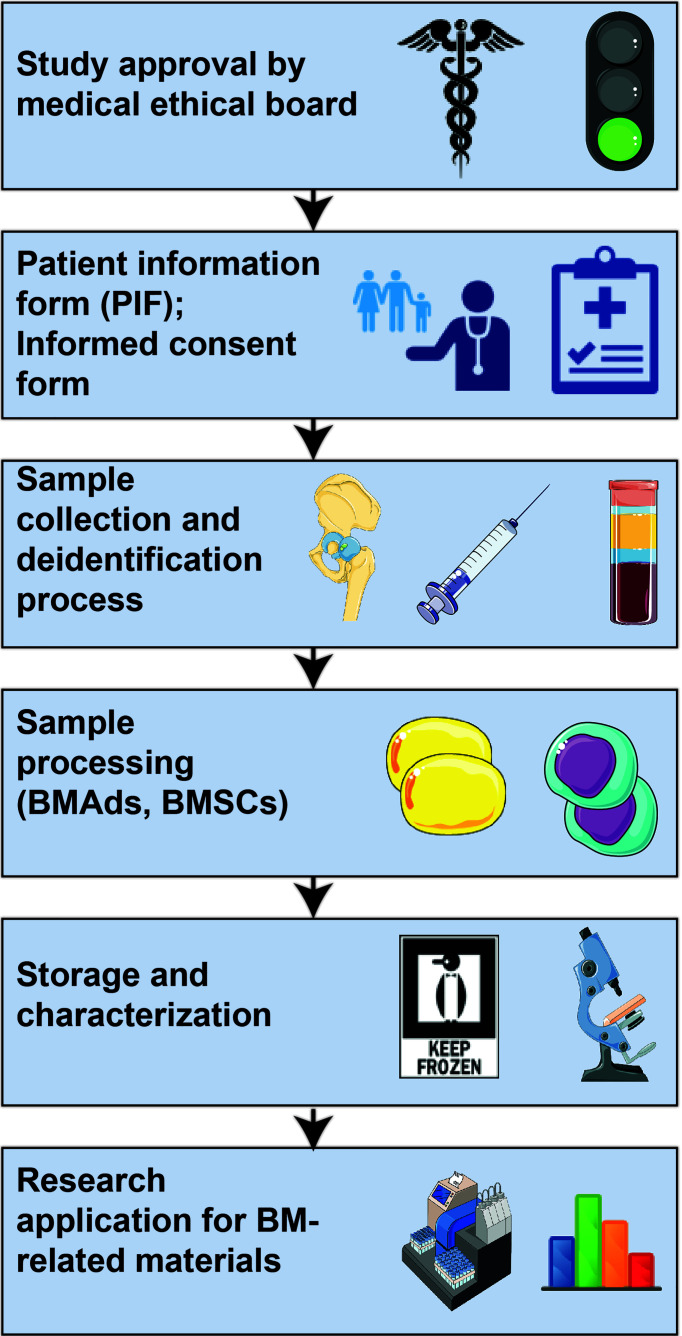
Workflow for BMA-related research.

The main goal, therefore, of this paper is to describe the current uniformities and discrepancies for the collection, use, storage and monitoring of BMA-related samples. A stepwise approach going from medical ethical approval through collecting and storing samples to the characterization and analysis of BMA-related samples is depicted in **Figure 1**. Although we are still far from a global harmonization of protocols, achieving this would greatly enhance the quality and interpretation of BMA-associated outcomes and increase the impact of BMA research. In fact, broad consent for future research may facilitate harmonization as well and, as it is ethically valid, this should be recommended for biobank research (9).

### 1.1 Introduction to Biobanking

Biological collections and collection-based science was first described by Carl von Linné in the 18^th^ century when he proposed the first system of classification for all biological species on earth (10, 11). The term “biobank” was used for the first time by Loft and Poulsen in 1996 when they suggested the use of human biological material to study a risk factor in cancer (12). Ever since, the field of biobanking has been expanding and it is now a fundamental and indispensable infrastructure for global health research (13–15). However, a BMA biobank has not yet been created. In general, a “biobank” includes large collections of biological samples such as DNA, cells, solid tissues or blood samples (16, 17), which are often stored for a long period of time and used only once a certain number of samples have been collected. They can also be processed, distributed, and used immediately. Samples are used by a variety of research projects and end-users, for example in population-based cohorts for diagnostic or interventional clinical trials, for medical needs/treatments, or for basic researchers validating their findings in human samples. Biobanks can be hospital-, government- or academic-based; networked or more isolated; and for profit or non-profit organizations (16–18). The population-based UK Biobank, between 2006 and 2010 collected biological samples from 500,000 volunteers without specific inclusion/exclusion criteria. Its aim was to investigate the influence of individual genetic susceptibility and exposure to external factors (19). In contrast, disease-oriented biobanks collect disease-specific specimens and non-diseased controls (20). Therefore, the definition of “biobank” varies greatly due to different types and components/purpose of biobanks, and because many biobanks evolved in a local and decentralized manner (13, 17, 21). Different national regulations established by local governance such as ethical guidelines or data protection provoke heterogeneity with regard to biobanking; however, for sharing biological samples with the scientific community, standard procedures for harmonization are required (21). Major advantages of the centralization of processing and standardization are the reduction of costs, increased throughput, and improved accuracy of sample handling and picking (22). The Organization for Economic Cooperation and Development (OECD) recommendations on Human Biobanks and Genetic Research Databases (HBGRD), published in 2009, provide guidelines for the establishment, management, governance, operation, access, use and discontinuation of human biobanks and genetic research databases (23). The structural key elements for sustainable international scientific infrastructure are named Biological Resource Centers (BRCs) as defined by the OECD (24). In addition, the International Organization for Standardization publishes standards for biobanks and bio-resources in ISO 20387 (ISO 20387:2018 “Biobanking – General requirements for biobanking”) to harmonize the biobank procedures. It also provides a professional environment for handling not only biological samples but also animal, plant and microorganism resources. Besides quality control of samples, ISO 20387: 2018 also includes validation of methods and competences of personnel (21, 25). In addition to the biobank infrastructure itself, the term “biobank” also covers bioethical and legal issues including ownership, informed consent and privacy – anonymization (17).

## 2 Informed Consent Form for BMA-Related Research

### 2.1 Providing Information Before Donation

Informed consent is a first, critical step in any tissue donation. Informed consent is not merely a disclosure form confirmed by a signature, it must also promote participant’s understanding of the research project and emphasize the voluntary nature of participation (26). The informed consent form consists of two parts: the informed consent certificate (containing approval of participant) and the Patient Information Form (PIF). Generally, the patient/donor is presented with the consent form after they have familiarized themselves with the PIF (see below). However, some institutions administer both consent form and the PIF simultaneously. The consent form should state that the patient had time and the opportunity to consider the information contained in the PIF, to ask questions, and obtain satisfactory answers to those questions. The consent form should explicitly state that the risks and benefits of the study have been clearly explained to the participant. The forms can be broad or specific to BMA research; the advantage of a broad consent form is that the collected samples can be used for a number of research projects that do not have to be specified at the time of consent. This breadth may be particularly useful for BMA research, which spans numerous other research fields. On the other hand, formulating the consent form specific for BMA research might make it easier for a donor to agree to participate, as they will know precisely what their samples will be used for. In fact, there is not yet a consensus on the policy regarding consent issues (27). The potential use of biobanked samples for translational work that may involve industrial collaborations and eventual commercial applications should also be discussed, as this may require explicit consent. For this reason, some countries have opted to include on their informed consent template a clause specifying that the patient waives future commercial rights.

Participants should realize that they are signing the consent to allow the storage and use of their specific biospecimens and any data resulting from the research using those specimens. Thus, it should be clear that only approved research studies can gain access to a donor’s de-identified data and samples. Studies that utilize the specimens and/or data need to receive prior scientific and ethical approval by the relevant committees, such as the IRB (Institutional Review Board), and they need to fit within the general requirements of the biobank and align with the consent form.

### 2.2 Deidentification as Prerequisite Before Consenting

Deidentification of data is a critical component of any research study involving human subjects. The procedures that will be used to protect the privacy and confidentiality of the subject’s data need to be clear to the potential donor prior to consenting. Unfortunately, biobank participants are not always made aware of the confidentiality risks associated with participation, which is a major ethical concern (28). Therefore, the donor needs to clearly understand how records will be secured, who will have access to the identifiable data, and whether names or ID numbers will be used. The procedure for securing the file linking the codes to individual subjects needs to be explained and participants should be informed about these issues while discussing the informed consent (29). Not all institutions include a Data Privacy Statement as part of their consenting protocol, and it is critical that this procedure is standardized across all institutions participating in BMA biobanking.

### 2.3 Possibility of Withdrawal From a Clinical Study

The consent form should state that the donor can withdraw from the study at any point and the acceptable ways to withdraw (e.g., email, phone call, letter) should be indicated. It should be clearly explained that upon withdrawal, identifiable samples and the associated data will be destroyed, unless the data have already been used for research. This is in line with the guidelines of the International Bioethics Committee (UNESCO) stating that handling of data and biological samples should follow the wishes of the donor unless they are irretrievably unlinked, making it impossible to do so (30). Accordingly, the consent form should indicate that the code that enables re-linking the samples with personal information will be deleted and only the signed consent form and a copy of the withdrawal letter will be kept as a record. These steps will prevent information about the donor contributing to further research and analyses.

## 3 Patient Information Form as Basis for BMA-Related Biobanking and Ethics Discussion

Many research institutes have templates for patient information and informed consent forms, and The Research Ethics Review Committee of WHO provides general templates and recommendations that can be adapted to specific needs (31). Within a PIF, the participant taking part in the study should be accurately informed about the planned study, what material will be collected and how it will be stored and used for research purposes. Although not every detail of the study has to be provided, the donor should have a rough idea of what will be done with his/her material and know that the collected samples will be handled with the utmost care and adherence to the various regulations, including those according to the General Data Protection Regulation (GDPR). The consent process can be improved by using clear and simple language in the documents (26, 32). Discussions between investigator and patient are encouraged to improve participant comprehension and ensure consent to participate (32). Below is a list of items that the WG believes should be mentioned in the PIF and which will be elaborated upon briefly, point by point (**Table 1**).

**Table 1:** Issues to be considered in patient information and informed consent forms.

Item	Patient information form (PIF)(presented to the participant before donation)	Informed consent form (signed by the participant, after considering the information in PIF)
**a. Information about the planned study**	*1. What is the purpose of the study?* - Background information and simple description of the study (broad or specific to BMA research) - Clear description of goals and expected results will help to increase the participation rates - Study is approved by Research Ethics Committee/Institutional Review Board *2. What sample material will be collected?* - BM sample (tissues/cells) - Reference samples (eg. subcutaneous fat, blood) *3. How it will be collected?* - Sampling procedure (surgery/biopsy/aspirate) - Risks and disadvantages related to BM sampling *4. What the sample will be used for?* - Broad purpose (unspecified projects) or BMA-specific purpose - General risks related to the sample donation and storage (eg. storage of genetic information)	Donor has had enough time to familiarize him/herself to the information provided in the PIF Donor has had a possibility to ask questions after considering the information in the PIF Donor understands the risks and benefits of participation Risks - General risks of sample donation - BMA-specific risks (e.g. risks related to the BM sampling procedure), blood sampling, or surgery. Benefits - No direct health benefits or financial benefits - Indirect benefits *via* promoting BMA-related research (and e.g. development of novel therapies in the future) Donor provides information about his/her clinical condition - Current diseases and treatments - Past diseases and treatments - Age, metabolic status and lifestyle habits (according to the study)
**b. Sample and data storage**	*5. Who can access the biological samples and the data obtained?* - Only approved research studies (with ethical approval) may have access to the samples - Tissue/cells that is left over from this study will be stored for future research to learn more about BMA	Donor allows the use of 1) biological samples and 2) the data resulting from the research using these samples. - The permission to store unused samples for possible future research should be separately requested.
**c. Anonymization**	*6. What procedures will be used to protect the privacy and confidentiality of the data?* - Explain what personal data is collected and how the data is anonymized - Sample material (and the data resulting from these samples) cannot be traced back to any of the person-related data	Donor understands 1) what data is collected and 2) how the data will be secured (Data Privacy Statement). Donor understands he/she will not have later access to his/her own data as all data is deidentified.
**d. Withdrawal**	*7. How to withdraw from the study?* - Participation is always voluntary - Provide contact information for withdrawal from the study (email, phone number, website) - Explain how the samples and associated data will be destroyed after withdrawal: new data cannot be obtained and that existing data will be maintained in a non-identifiable form	Sample is donated to biobank voluntarily. Donor can withdraw from the study later at any time and without any reason. - Explain how the withdrawal should be signaled to the researchers (eg. email, letter, phone call)

### 3.1 Positive Disadvantages or Risks and Benefits of Donation

With every medical treatment or procedure there is some risk involved. Thus, the sample procurement procedure should be sufficiently risk-assessed and communicated to the donor. Standard donation through tissue biopsies or blood drawing constitutes minimal risk, while BM biopsies or aspirations are more invasive and have an increased risk of various complications that need to be specified. Complications are rare, but as with any procedure involving a tissue biopsy, there is a small risk of bleeding and/or bruising from the sampling site. Moreover, there are descriptions of rare cases of deep tissue infections, severe internal bleeding or bone fracture associated to the BM biopsy/aspiration procedure. In the case of sampling as a byproduct during knee/hip surgery, there are no other risks beyond those usually associated with surgery. In all cases, there should be a lead doctor or research nurse involved to answer questions surrounding the tissue/cell collection. Moreover, donors should be informed about the potential risk of not being able to use the material, for example by unanticipated loss or low quality of the material obtained.

Benefits also have to be addressed towards the donor, with the general message that no direct benefits (e.g. financial or material) can be obtained from participating in a study. Having said that, being a donor contributes to the general understanding of diseases or tissues, such as BMAT, which may lead to future development of new, effective treatments, and thereby may benefit patients. In addition, healthy control donors are often compensated for their involvement in a research study in the form of a small fee for the incremental inconvenience, burden or risk associated to dedicated tissue collection, as opposed to the often-uncompensated donation of residual tissue obtained upon routine medical procedures (33–35). Providing relevant background information about the goals of the study and the expected results is key and will increase participation rates. This will lead to more impactful studies with more reliable conclusions on BMA-related outcomes.

### 3.2 Constraints and Safeguards of Biobanking

For any clinical study to start, two critical steps are required. Firstly, approval of a medical ethical board is required within the institute where the study is conducted. When a study is multi-centered, there should be an additional approval from the cooperating institutes, but this could be partial or less stringent as long as the coordinating center has a full approval.

Secondly, a valid, written consent is needed before the acquisition or use of the intended BMA-related sample or associated clinical data. By definition, the donor’s participation is always voluntary and he/she can always withdraw from the study. The participant will never have access to his/her own data as all data are anonymized. In fact, as soon as a sample is obtained, it should be fully deidentified through encoding in any biobank. None of the users employing the samples or analyzing data belonging to the study can trace back any of the material to any of the person-related data belonging to the donor. This is one of the primary requirements for any medical study involving patient or participant biomaterial and all effort should be taken to prevent any breach of confidentiality.

### 3.3 Differences Between Institutional PIFs and Biobanking Procedures

In preparation of this position paper, the BMAS Biobanking WG has scrutinized and compared PIFs derived from seven different institutes across Europe and the US. It became evident that there are differences regarding the various aspects described above, especially in the context of the actual content and the level of detail of the proposed research, but also with respect to which measures have to be taken to safeguard the anonymity of the participant in the study and the protection of the acquired data. We thus realized that there are many hurdles to overcome in an attempt to harmonize protocols and procedures across the globe.

## 4 Biological Materials Relevant for BMA-Related Research

The types of tissue/cell that will be collected for BMA-related studies are primarily driven by the sample accessibility. Bone and BM samples can often be obtained during invasive procedures, such as surgeries (joint replacement, amputations, open-heart or spinal surgery), autopsies, BM biopsies and BM aspirates (8). The nature of the research depends on the surgery and biopsy type, which determines the anatomical site, patient characteristics (underlying pathology, age, sex, etc., as reviewed below), quantity and quality of sample, and transportation/storage capabilities. Other tissues, such as subcutaneous white adipose tissue (WAT) and venous blood, can also be collected in parallel, but only waste/discarded material, or small samples that cause minimal risk, should be used. Subcutaneous WAT is relatively easily obtained at the site of surgery and can be used as a reference for BMAT analyses, and serum/plasma can be assessed for relevant biomarkers, for example for metabolic function and bone remodeling (biochemical analyses).

**Figure 2:**
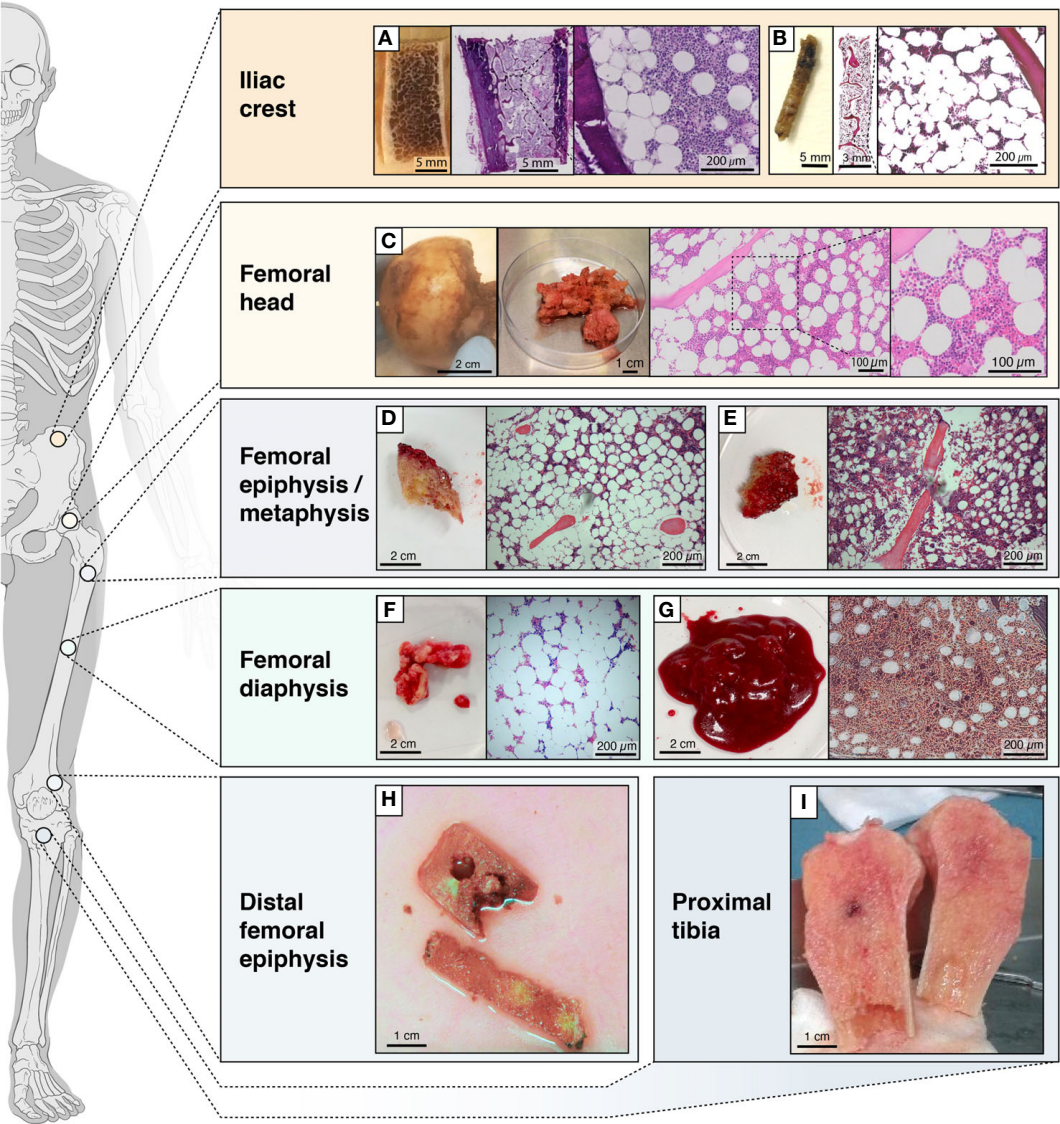
Schematic figure showing the locations from which BMAT is obtained. Locations from which BMAT is obtained, heterogeneity in tissues (e.g. more fatty *vs* less fatty) and collected fractions. **(A)** transiliac bone autopsy obtained with a Bordier trephine and **(B)** iliac bone marrow biopsies obtained with a Jamshidi trephine; **(C)** femoral head autopsy; **(D)** epiphyseal or **(E)** metaphyseal tissue from femur; **(F)** diaphyseal bone or **(G)** bone marrow from femur; **(H)** tissue from the distal femoral epiphysis; **(I)** bone tissue from the proximal tibia.

The presence of BMAds is variable depending on the surgery site but could be also due to patient heterogeneity (**Figures 2A–I**). For example, as demonstrated from Magnetic Resonance Imaging analyses, the femoral head is expected to be more enriched for BMAds compared to the ilium, while the femoral or tibial diaphysis are expected to have even greater BMA than the femoral head (36). The pathophysiological context can also be a source of heterogeneity in BMAd development and phenotype, and can consequently influence the BMAd isolation, analysis, and study outcomes. Indeed, age, sex, lifestyle habits, metabolic status (e.g. obesity, diabetes, anorexia), medications (e.g. thiazolidinediones, glucocorticoid analogs, hormonal substitutive therapy, radio- or chemo-therapy) and the pathology underlying the orthopedic surgery (osteoporotic fractures, osteoarthritis, osteonecrosis, etc.) are conditions known to modify the BMAd component [as reviewed in (37–41)]. Therefore, it is important to collect donor clinical characteristics, including age, sex, body mass index (BMI), medication history, and incidence of fractures in order to better define the heterogeneity of clinical material (**Table 1**).

In this context, it is very challenging to establish a harmonized “healthy” control set for biobanking as the definition of “healthy control” varies among different studies, depending on the clinical sampling, the aim and biological questions. The “healthy control” samples are ideally obtained by BM biopsies and aspirates from healthy volunteer donors. Yet, as emphasized above, ilium may not represent the most reliable bone site to study BMAds. Orthopedic surgery of healthy patients (not diagnosed with BM diseases or necrosis) following trauma or amputation are an exception, while it is sometimes possible to get “healthy” samples considered as debris during surgery. In fact, this type of surgery currently delivers most of the “healthy” samples. Post-mortem sampling from organ donors can also be considered if the subsequent analyses (such as adipocyte histomorphometry) are compatible with a delayed processing; however, BMAd molecular characteristics are likely to be compromised in such post-mortem samples. It is the responsibility of the research team to clearly establish and describe the inclusion and exclusion criteria, which subsequently allows for categorization of “healthy” or “control” *versus* “study” group(s). Alternatively, a referent cell type (e.g. subcutaneous adipocytes, BMSCs from another bone site) isolated from the same patient can be used for comparisons.

### 4.1 Choice of BMA Relevant Biological Materials and Their Use

Due to the various types of tissue and cell types relevant to BMA, the unclear definitions of cell populations and the relatively recent emergence of the field, the methodology behind collecting BMAT, BMAds and BMSCs is heterogeneous as recently reviewed (8). It is important, for all samples that have been collected, to specify if screenings have been performed for viral diseases (typically HIV, HBV and HCV), as in some countries a negative test is a prerequisite to allow usage of the samples in research facilities. So, for the future of biobanking it is important to keep track of these variables.

#### 4.1.1 Operation Specimens and Autopsies

A reliable method to obtain fresh BMA-relevant biological material is during orthopedic surgeries, such as knee and hip replacements, reconstructions, corrections and amputations. During these operations, a key part of the surgical procedure is removal of bone and BM that is often discarded after the surgery. It is possible to recover this biological material for BMA-related research and biobanking. A good example is hip replacement surgery, where the femoral head and part of the trabecular bone cavity are removed and simply discarded, if not transferred to a bone bank as allograft material. With these surgical specimens, it is possible to process samples for downstream detailed histology (see BM biopsies) (**Figures 2A–I**) or to isolate almost all desired adipocytic, stromal and hematopoietic BM cell types; see also section 4.2.

Post-mortem autopsies offer a unique opportunity to obtain BMA-relevant biological materials from several skeletal sites, and not only the classical sites undergoing orthopedic surgery or BM biopsies. As mentioned earlier, these autopsies are useful only for simple adipocyte histology and histomorphometry, and ex vivo imaging by micro-computed tomography (µCT); they are not suitable for molecular analysis and cell isolation (unlike for fresh operation specimens and BM biopsies). Nevertheless, histomorphometry and imaging of autopsy samples are still useful as they allow for analysis of the BM compartment to analyze the BMAd content, morphology and size (**Figure 2A**). High quality tissue may however be banked in the exceptional context of systematic bone tissue collection from post-mortem organ donors.

#### 4.1.2 Bone Marrow Biopsies

Transiliac bone biopsies obtained with a Meunier/Bordier trephine (inner diameter 5-8 mm) have for decades been the gold standard within bone research and diagnostics. These biopsies are obtained under a quite invasive procedure, usually performed under general anesthesia. For BMA-related research, it is sufficient to obtain iliac BM biopsies with a Jamshidi trephine [inner diameter 3 mm, recommended length 2 cm (42)]; this is a much-less-invasive procedure usually performed under local anesthesia, which also allows for collection of a BM aspirate (**Figure 2B**). These 3-mm bone marrow biopsies are the gold standard in hematology research and diagnostics, and due to their less-invasive nature they are more justifiable from an ethical perspective. The usability of these biopsies is versatile for BMA-related research, as it is for surgery specimens. For BMA-related research by histology there is no need to embed the undecalcified specimens/biopsies in plastic, as done for classical bone histomorphometry. Instead, the specimens/biopsies can be fixed, decalcified and paraffin- or frozen-embedded. *Ex vivo* imaging by µCT or similar method can be performed before the specimens/biopsies are decalcified. The embedded specimens/biopsies are then usable not only for adipose histomorphometry (see autopsies), but also for more advanced molecular histology, like multiplex immunostaining, *in situ* hybridization, laser microdissection and spatial transcriptomics (8). This facilitates detailed analysis of BMAd spatial distribution, gene expression profile, and interaction with other cell types in the BM.

#### 4.1.3 Bone Marrow Aspirates

BM aspirates are primarily taken from the iliac crest or femoral BM biopsy (occasionally from femoral epiphyses) site, usually following the biopsy under local anesthesia. This is a less-invasive procedure and allows for relatively easy collection of BM fluid containing various cell types, including adipocytes (43–45). The BM aspiration procedure is not necessarily familiar to the participant and the PIF should describe what happens during sampling on a step-by-step basis, including injection of the local anaesthetic into the location where the sample is to be taken from. BM aspirates are especially useful for the collection of various cell types, including a BMAd-enriched fraction (see *Section 5*) and BMSCs (8). The cells can be directly cultured following collection, or they can be prepared for storage to be analyzed later. Typically, the different BMSC-related cultures are employed to study cell behavior, differentiation, marker expression and/or their interaction with other cell types (mimicking the situation in BM). BMAd-enriched fractions can be extemporaneously used or stored for further molecular and biochemical analyses. Although no cell type or tissue collection is involved, *in vivo* analyses of BM are increasingly available and provide a good source of information on BMA content, composition, spatial and temporal distribution (etc.), and it would be very helpful to compare these *in vivo* analyses to biobank data (8).

#### 4.1.4 Subcutaneous Fat

Subcutaneous WAT can be collected at the surgery site and provides a rich source of adipocytes and Adipose tissue-Derived Stromal Cells [ADSCs, nomenclature according to (7)], that can be used for reference, for example for comparison with BMAds and BMSCs.

#### 4.1.5 Blood Samples

Venous blood can be collected in different ways depending on the research question but most often is stored in the form of serum (for measurement of biochemical parameters e.g. lipids, growth factors, hormones or plasma collected in the presence of anti-clotting agents (e.g. EDTA or Heparin) (for molecular analyses, chemistry, or cell culture). Quantification of metabolites in blood samples requires standardization of pre-analytical processing and rapid cooling down, as reviewed here (46–48). If blood samples are obtained in the fasted state, it is also possible to investigate the level of bone resorption and formation using markers like P1NP, CTX, NTX and TRAcP, and link this to the bone metabolic state (49).

### 4.2 Isolated Human Primary BMAds and BMSCs

BMAds are present scattered or more packed within the BM of different bones in humans (**Figures 2A–I**). Accessibility of the BM cavity is obviously required to isolate BMAds, which restricts the bone sites available to obtain samples [i.e., BM biopsy sites, resected bone pieces and BM aspirates of long bones obtained during orthopedic surgery (**Figures 2A–C, 2E** and **2G**)]. So far, BMAds and BMSCs have been obtained from different locations including the femoral head (50, 51), the diaphyseal end of the femur or tibia (52), BM aspirates from the femur (53) and the iliac crest (54, 55) (**Figures 2A–C, F–G, I** and **4**).

Specific procedures for the collection of BMAds are relatively novel (*see below*), and in some cases freshly isolated BMAds may be needed for immediate analysis. However, BM fragments and BMSCs have been successfully collected and biobanked, followed by optimized characterization of the cryopreserved material (56, 57).

The process of sampling is also variable and influences the quality of BMAd isolation. For example, for hip arthroplasty the femoral head is removed using a surgical saw and, prior to insertion of the artificial hip, the medullary canal is cleared using a reaming tool before aspiration of the BM. These steps are often done using electric saws and reamers, but non-electric tools can also be used. Similarly, some electric reamers simultaneously aspirate the BM cavity, but aspiration can also be done manually, post-reaming, using a syringe and soft cannula. These different methods (i.e. electric *vs* non-electric) can affect the quality of BMAds as a result of differences in heat production and/or mechanical stress (51, 53, 58).

Thorough discussions with the surgeons are thus instrumental to determine the optimal processes and to adapt surgical techniques, when possible, for BMAT sampling.

As previously encouraged (8), a detailed description of the source of BMAds is consequently required for biobanking purposes and study comparisons. Firstly, minimal information should state the original skeletal location, i.e. distal/proximal bone site, trabecular bone or BM (**Table 2**). Secondly, patient characteristics should be reported including the distribution by age, sex and BMI as well as the pathophysiological context, i.e. the presence of bone disease, osteo-articular disease, hematological disease, malignancies, and their related treatment. According to the studied questions, other specific parameters (such as BMI, metabolic status when relating to energetic metabolism perturbations for example) are also expected. Thirdly, how the original clinical sample is obtained (i.e., use of electric or non-electric surgical tools, use of digestion or other sample processing and any other relevant details) should also be described.

**Table 2:** Summary of critical steps for BMAd and BMSC isolation to highlight differences between protocols.

Main steps	EDIN	TOUL	LILL	LAUS	PRAG
***Material***					
**Bone site of sampling**	Femoral proximal metaphysis and diaphysis Femoral head	Femoral proximal metaphysis and diaphysis	Trabecular bone from distal femoral epiphysis	Femoral head (epiphysis + variable quantity of metaphysis) Iliac crest	Iliac crest
**Sample type**	BM aspirates	BM aspirates	Cancellous bone	BM aspirates	BM aspirates
**Final material obtained**	BMAds	BMAds	BMAds	BMSCs and BMAd-enriched fraction	BMSCs and BMAd-enriched fraction
***Digestion***					
**Buffer**	KRH, 5.5 mM glucose 3% BSA	PBS 2% BSA	DMEM, 5.5 mM glucose 3% BSA	DMEM, 10% FBS	**-**
**Collagenase type, manufacturer (reference number)**	Type 1, Worthington Biochemicals (LS004196)	from *C. histolyticum*, Sigma Aldrich (C6885)	NB 4 standard grade, SERVA Electrophoresis (17454)	Type I, Gibco (#17100-017)	**-**
**Collagenase concentration**		250 UI/mL	0.2 to 0.3 UI/mL	1 UI/ml	**-**
**Digestion time**	45min	Max. 20 min	15-30 min	45 min -1h	**-**
***Washing***					
**Buffer**	KRBH 5.5 mM glucose	KRBH 0.5% BSA	DMEM, 5.5 mM glucose/ 3% BSA	PBS/1% BSA	PBS/1% BSA

Different protocols have been set up to isolate BMAds (from 3 institutes: EDIN, TOUL, LILL) or BMSCs (from 2 institutes: LAUS, PRAG). Differences related to biological material, digestion and washing parameters are highlighted.

Abbrevations referring to institute cities of members within BMAS Biobanking WG. EDIN: Edinburgh (WC); TOUL: Toulouse (CA); LILL: Lille (SL), LAUS: Lausanne (ON); PRAG: Prague (MT).

## 5 Methodology of BMA-Related Cell Isolation and Analysis

Various protocols for human BMSC isolation (55–57, 59–61) and, to a lesser extent, for BMAd isolation (50–54, 62–64) have been published. Isolated BMSCs are classically used *in vitro* to analyze their differentiation capacity into different lineages, to study BM cell interactions, to characterize progenitor markers, to analyze BMSC senescence and to compare their molecular and cellular characteristics to different sources of stem cells, including peripheral ADMSCs. Thus far, applications for isolated BMAds are devoted to characterizing their specific phenotype by comparison with extramedullary adipocytes, such as subcutaneous WAT adipocytes, using RNA analyses, proteomics or lipidomics. The downstream analyses, as well as the research context encompassing the biological questions, the clinical sampling and the type of comparative/referent samples, have to be taken into consideration for the choice of the isolation method.

**Figure 3:**
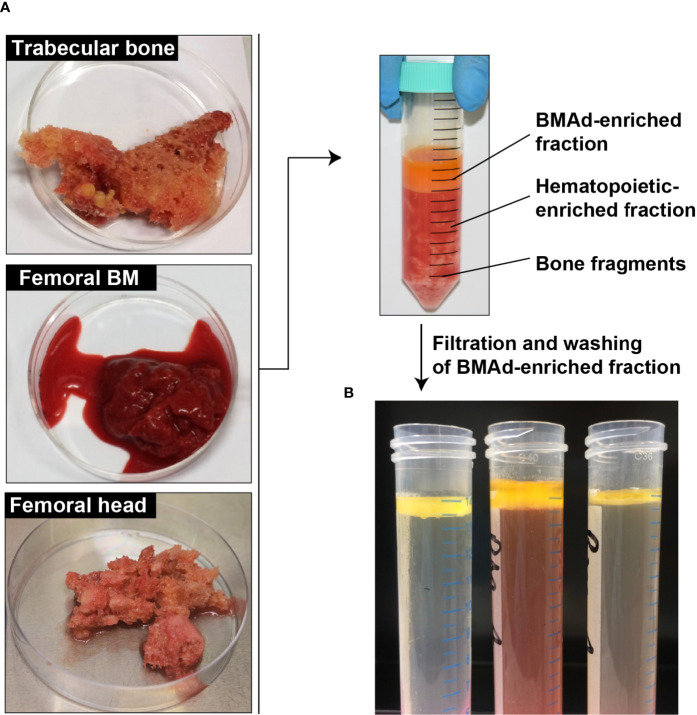
Processing of the BMAd-enriched fraction without digestion step. **(A)** Samples are received from surgeries into their transport buffer. The adipocyte-enriched layer is harvested, filtered and washed. **(B)** Several examples of BMAd-enriched fractions after washing are shown.

**Figure 4:**
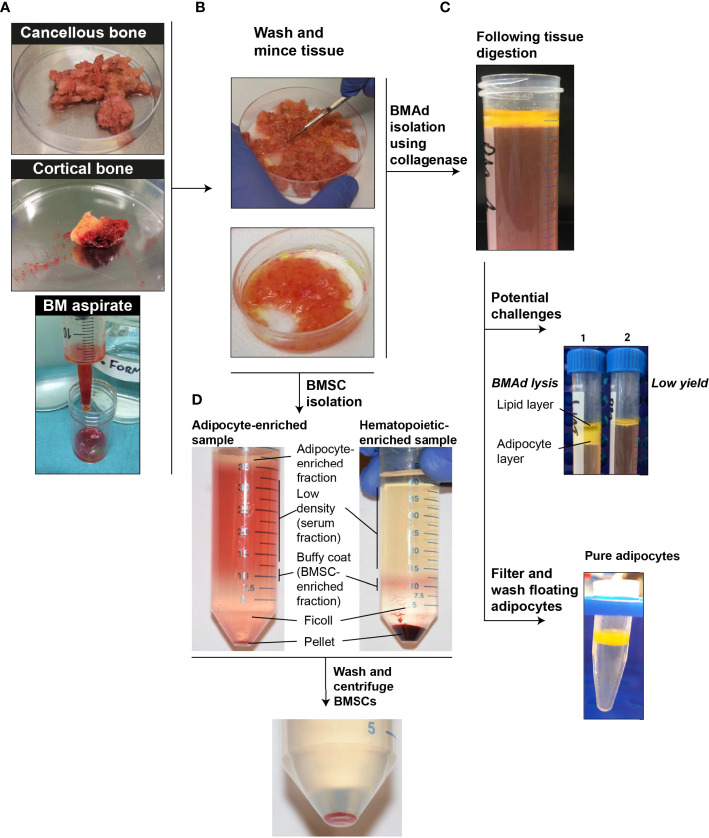
Schematic protocol for isolation of BMAds and BMSCs. **(A)** Strategy to recover BMAds and BMSCs depending of the tissue source. **(B)** Wash and mince the selected tissue. **(C)** Digestion strategy using collagenase digestion to increase the release and the purity of BMAds. Some encountered challenges are shown such as the lysis (1) and low yield (2) of BMAds. At the end of the process pure BMAds are obtained. **(D)** BMSCs isolation with tubes showing the variation observed between samples. BMSCs are obtained after centrifugation of the serum fraction and the BSMC-enriched fraction.

In this review we provide dedicated protocols for isolation of BMAds and/or BMSCs. As discussed within each section, the absence of other contaminant cells, the cell integrity and the cell yield determine the success of the method. Considering the great heterogeneity in patients and samplings, it is advised to file a small piece of the initial tissue for histological analysis (**Figure 2**). Besides, BMSCs can be obtained, though in small amount, during the BMAd isolation procedure. In addition, a floating lipid-loaded cell layer is often observed following the processing of BM aspirates or trabecular bone samples before any collagenase digestion (**Figure 3A**). This cell fraction most likely results from the surgical procedure that allows the release of BMAds that are loosely attached and contains both BMAds and contaminant cells from the hematopoietic and the stromal compartment. We suggest referring to this as a BMAd-enriched fraction (i.e. fraction that is obtained from BM but without collagenase digestion, as illustrated in **Figure 3B**), which could be used for lipid analysis. Yet, this BMAd-enriched fraction should be used only after digestion and washing steps if pure BMAds have to be analyzed. This floating lipid-loaded cell layer is not to be confounded with the free lipid layer that results from spontaneous cell lysis upon excessive mechanical processing, excessive ambient temperature or handling time (**Figure 4**).

### 5.1 Isolation of BMAds

Various laboratories have already started working on BMAds isolated from BM aspirates, from femoral diaphysis (51–53, 64), from trabecular bone of the proximal femoral metaphysis/epiphysis (51), the femoral head (50, 62, 65), femoral BM fluid (62), the proximal tibia (52) or the iliac crest (54) (**Figures 2A–I**). Within our working group, three protocols were set up and validated to isolate and purify BMAds, and accordingly, the critical steps to obtain pure primary BMAds for different analyses are depicted here. These main steps are based on protocols commonly used for the isolation of adipocytes from peripheral WAT, which have been adapted from the initial work of Rodbell (66). Tissue digestion is commonly performed using collagenase to release BMAds from the extracellular matrix, which is mainly composed of collagen fibers (67). Indeed, if the study relies on a comparison between isolated BMAds and WAT adipocytes, a similar method based on collagenase digestion should be performed. However, this enzyme-based isolation is not required if the study focuses on characterizing only BMSCs. Within the following sections we describe how the clinical sample is obtained and provide a general protocol to process these samples with joined notes to discuss key aspects. The main differences between our three protocols relate to reagents, as highlighted in **Tables 2**, **3**. It is important to emphasize that each protocol has been tested and established separately. As combining reagents between the protocols has not been validated, it is recommended to strictly use the advised reagents for the selected protocol.

**Table 3:** Composition of buffers used for sample transportation.

	EDIN	TOUL	LILL	LAUS	PRAG
Buffer name	KRH	KRBH	DMEM	PBS 1X + EDTA 1 mM	MEM
**MgCl_2_; 6H2O**	N/A	0,5	N/A	N/A	N/A
**MgSO_4_**	0,6	N/A	0.8	N/A	0.8
**KCl**	2	4,6	5.4	2.7	5.3
**NaCl**	120	120	110	137	117.24
**Na_2_HPO_4_ (anhydrous)**	N/A	0,7	N/A	10	N/A
**NaH_2_PO_4_ (anhydrous)**	N/A	1,5	1	N/A	1
**KH_2_PO_4_**	1	N/A	N/A	1.8	N/A
**CaCl_2_; 2H_2_O**	1	N/A	1.8	N/A	1.8
**D-Glucose**	5,5	10	5.5	N/A	5.5
**NaHCO_3_**	N/A	150	44	N/A	26.2
**HEPES**	81,6	10	N/A	N/A	N/A
**Bovine Serum Albumin (BSA)**	1%	0.5%	N/A	0	N/A

Five different buffers have been used for sample transportation: Krebs Ringer Bicarbonate HEPES buffer (KRBH), Krebs Ringer HEPES (KRH) and Dulbecco’s Modified Eagle’s Medium (DMEM, Dutscher, L0064). The concentrations of the different components are indicated in mM, except for BSA.

Abbreviations referring to institute cities of members within BMAS Biobanking WG. EDIN, Edinburgh (WC); TOUL, Toulouse (CA); LILL, Lille (SL); LAUS, Lausanne (ON); PRAG, Prague (MT).

#### 5.1.1 Isolation and Transport of Samples

As mentioned above, BMAds are isolated from femoral head, BM aspirates of femoral proximal metaphysis/diaphysis or trabecular bone from distal femoral epiphysis (**Figure 4**).

The femoral head is removed with an electric bone saw and stored in buffer or processed immediately in the surgical theater and the bone fragments obtained by mechanical processing stored in transport buffer. To obtain BMAds from the femoral head (51, 65), the trabecular core is first exposed by bisecting the femoral head longitudinally using an electric bone saw. A sterile spoon spatula is then used to cut and scoop out ~1 cm^3^ portions of the trabecular bone from within the femoral head. The medullary cavity of the proximal femoral metaphysis and diaphysis is then cleared of trabecular bone by using a manual reaming tool, with a bone mallet used to drive the reamer into the medullary canal. To obtain BMAds from it, portions of trabecular bone are then removed from the reaming tool, washed and stored in buffer (51) (**Figures 4A, B**).

To obtain BMAds from the proximal diaphysis, BM from this region is aspirated using a soft cannula attached to a manually operated syringe. The aspirate is then transferred to a sterile petri dish or specimen tube prior to washing and storing in buffer (51, 53, 64) (**Figures 4A, B**).

To obtain trabecular bone from distal femoral epiphysis, 1 cm^3^ of cancellous bone square is taken in the center of the femoral epiphysis using adapted cold chisels after the first distal femoral cut during knee prosthetic surgery (**Figures 2D–H**).

As soon as the sample is harvested, it must be placed in transportation buffer (**Table 3**) and transferred to the laboratory in the quickest possible manner**^1,2,3^**.

***Notes:***

*1- Different buffers are used in our laboratories and the composition of these buffers is detailed in* **Table 3** *. All these media should be at pH 7.4 (buffered with HEPES and/or bicarbonate) and contain inorganic salts and glucose as nutrients are needed to maintain tissue integrity.*
*2- It is recommended to transport the sample at room temperature (~20°C) and to minimize strong temperature changes. Yet, for some applications such as molecular analysis (RNA expression, protein or lipid content characterization), keeping samples at 4°C (on ice) can be advised as reported in* (51)*. For biobanking purposes, the effect of transport/storage conditions on cell properties and on any post-isolation changes still remains to be tested. At the moment, we recommend documenting the transport/storage conditions for every sample.*
*3- Ideally the transportation should be achieved within 30-60 minutes since the reduction of oxygenation and nutrient deprivation at the center of the sample may compromise tissue integrity, as already reported for other adipose tissues* (53, 64).

#### 5.1.2 General Protocol for BMAd Isolation

**Reagents and Equipment:**

- digestion buffer with collagenase (composition in **Table 2**), pre-warmed at 37°C
- washing buffer (composition in **Table 2**), pre-warmed at 37°C
- shaking water bath at 37°C
- Polypropylene tubes**^4^**
- cell strainer (100 µm) or a Nylon mesh (porosity between 150 to 300 µm)
- classical instruments and lab pipets/tips

***Notes:***

*4- To limit the lysis of BMAds we recommend working only with plastic, because glass tubes cause lysis of adipocytes.*

**General Protocol (** **Figures 4B, C** **)^5^:**

Rinse the tissue several times in the transport buffer (**Table 2**) to remove tissue debris and clotted pieces.
Mince the tissue in small pieces to facilitate digestion**^6^** and add tissue pieces in the digestion buffer**^7,8^** containing collagenase**^9^**.
Digest the tissue at 37°C in a water bath under gentle shaking (from 120 to 200 rpm) with a careful and continuous monitoring of the incubation time**^10^**.
At the end of digestion**^11^**, filter the cell suspension, either on a cell strainer or a Nylon mesh over a tube. The strainer or mesh should have a pore size of at least 100 µm, given the large size of adipocytes.
Add washing buffer to dilute the collagenase and to rapidly stop the collagenase action.
Allow the adipocytes to then float in the filtered suspension either storing upright the tubes or centrifuging (5min, 300 g).
Aspirate the infranatant and the cell pellet**^12^**.
Wash 3 times the floating adipocytes by adding washing buffer and centrifuging for 5 min at 300 g to pellet contaminant cells**^13,14,15^**.
Collect the BMAds in this floating layer using a micropipette**^16^** or by removing the remaining washing buffer as much as possible following a short centrifugation step to pack the floating cells**^17^**.

***Notes:***

*5- The isolation protocol is performed at room temperature with the exception of the digestion step. Please be aware that in poorly temperature-controlled environments (for example in the summer months), BMAd viability may be considerably compromised.*
*6- Unless the tissue is already partly dislocated, such as for the BM aspirates, the other tissue types (e.g. cancellous bones) are then minced in small pieces to facilitate digestion. Cancellous bone from the femoral epiphysis or metaphysis is for example transferred in a small flat plastic dish and kept immersed in digestion buffer to be cut using a scalpel or scissors to get pieces of ~5mm^3^. During cutting, the bone can be held in place using cutting clamp or forceps, as needed.*
*7- The digestion is favored by an appropriate balance between the volume of digestion buffer and the amount of tissue fragments. We recommend the use of two buffer volumes for each sample volume or, if preferred, 2 mL buffer per gram of tissue.*
*8- Digestion buffer can vary from PBS, Dulbecco’s Modified Eagle’s Medium (DMEM) to Krebs Ringer HEPES (KRH), highlighting the importance of a basic ionic strength (* ***Table 2*** *). The presence of glucose may not be necessary during a short digestion duration but can be advised to minimize changes following transportation or when metabolic features of BMAds are studied. Importantly, the presence of fatty-acid-free Bovine Serum Albumin (BSA) at classical concentrations of 2 to 3% (w/v) is instrumental: BSA binds the released fatty acids and prevents their detergent action on cell membrane* (68).
*9- Different collagenases can be used with yet a careful choice (* ***Table 2*** *). Indeed, collagenase preparations differ between manufacturers and from batch to batch. Thus, our labs first check the efficiency of the collagenase batch they use and determine the collagenase amount and the digestion duration to obtain a BMAd suspension. BMAds are more fragile and loosely associated compared to adipocytes from WAT. Thus, digestion time for BM tissue can be reduced; however, it remains unclear how differences in digestion duration affect the final properties of the BMAds and WAT adipocytes.*
*10- An optimal shaking should provide the appropriate diffusion of the collagenase with the whole tissue fragments while mixing the fatty acids with BSA, and yet should also avoid BMAd lysis. This shaking can modulate the digestion duration and can also be adapted according to the samples. As a general rule, shaking force should be highest at the beginning and reduced with time. Incubation time can vary between 15 and 45 minutes.*
*11- Digestion occurs when increased turbidity appears. The medium becomes more and more opaque because of BMAds which, once released, float at the surface (especially evident once the tube is no longer being shaken). This step should be continuously monitored and stopped when cell release from bone pieces seems reduced. Besides, the digestion must be stopped when large amounts of lipids are released (i.e., oil appearance in the suspension meaning that adipocytes burst).*
*12- The cell pellet contains BMSCs, which can be harvested at this step. After removing the adipocytes, the remaining buffer with collagenase is aspirated. In a sterile hood, pellet is resuspended in red blood cell lysis buffer according to the manufacturer’s instructions: add 1ml of buffer, vigorously pipette to mix, let sit for 1 min, add 15 ml of KRH buffer (without BSA), pipette to mix. Then, split cell resuspension into two 14 mL falcon tubes and centrifuge at 300 g for 10 minutes prior to remove the supernatant. Then the pellet is resuspended in appropriate culture medium as described in the BMSC section (5.2.3).*
*13- Even after cell dissociation with collagenase and separation of adipocytes by flotation, some contaminant cells can remain attached to adipocytes. Thus, to purify adipocytes, it is important to wash and centrifuge the adipocyte suspension to pellet contaminant cells.*
*14- BMAds are fragile and can burst during the washing steps so we recommend doing this step as quickly and gently as possible.*
*15- For Western blots or proteomic analyses, BSA must be removed to allow a correct protein quantification of the samples. To do so, it is important to wash twice with PBS.*
*16- If using a pipette, it is recommended to cut the tip using a scalpel to increase the bore size of the pipette tip; this prevents shear stress and lysis of the BMAds.*
*17- If BMAds are to be used only for downstream molecular analysis, it can be advised to store the tube on ice to facilitate formation of the floating adipocyte layer, to use ice-cold washing buffer to minimize any further molecular changes and to centrifuge at 4 °C. In that case, the use of glass pipettes to homogenize in the RNA extracting reagent is beneficial because this promotes BMAd lysis and prevents adherence of the cells to the pipette, thereby increasing yields.*

#### 5.1.3 Freezing and Storage of BMAd Samples

The isolated BMAds can then be frozen according to the future analyses^18^ as indicated in the overview in **Table 4**. Special attention should be paid to the cell volume to adapt the volume of RNA extraction reagent (about 1:6 or 1:10 v/v) and to remove lipids. As for adipocytes from WAT, excessive lipid can prevent efficient RNA isolation. Thus, following initial cell lysis, it is helpful to allow the homogenate to settle on ice, resulting in a formation of a floating lipid layer. This lipid layer can then be removed and discarded, to be replaced by a similar volume of RNA extraction reagent.

**Table 4:** Recommended storage of isolated BMSC and BMAd samples.

Storage of the samples	BMSCs	BMAds
**Cell cryopreservation**	One million cells in freezing media (per vial: 80% FBS, 10% DMSO, 10% MEM or 50% MEM, 40% FBS, 10% DMSO) in liquid nitrogen).	Impossible to keep viable frozen BMAds. Frozen isolated BMAds can be snap-frozen in liquid nitrogen and stored at least at-80°C for further analyses.
**RNA**	In Trizol (or similar) at -80°C	In Trizol (or similar) at -80°C
**Protein**	In protein lysis buffer at -80°C	In protein lysis buffer at -80°C
**Lipids**	Flash freezing in liquid nitrogen within 4 hours after surgery. Store at -80°C	Flash freezing in liquid nitrogen within 4 hours after surgery. Store at -80°C

For untargeted lipidomics, BMAds can be frozen in methanol to allow a better stability of lipids. For targeted lipidomics, the extraction buffer is different depending on lipid classes. Thus, we recommend freezing samples directly in the appropriate extraction buffer. In other cases, BMAds can be frozen without extraction buffer but under nitrogen gas to prevent the oxidation of lipids.

***Notes:***

*18- We recommend freezing a known volume of adipocytes in liquid nitrogen and keeping samples at -80°C until use. When subsequent analyses are already planned, samples can be stored in specific media for better stability.*

#### 5.1.4 Approaches to Check Purity and Viability

One issue with BMAd isolation is that non-BMAd populations may contaminate the layer of floating cells. As an example, human BMAds can have direct interactions with osteoclast precursors (62). Considering that macrophages are contaminants of isolated WAT adipocytes, other surrounding cells (e.g. BMSCs, endothelial cells, hematopoietic or immune cells) may remain attached to the floating BMAds. In addition, the evaluation of BMAd viability may be beneficial to discard cell alteration from the isolation procedure or for subsequent functional analysis.

##### Evaluation of Cell Purity

**Figure 5:**
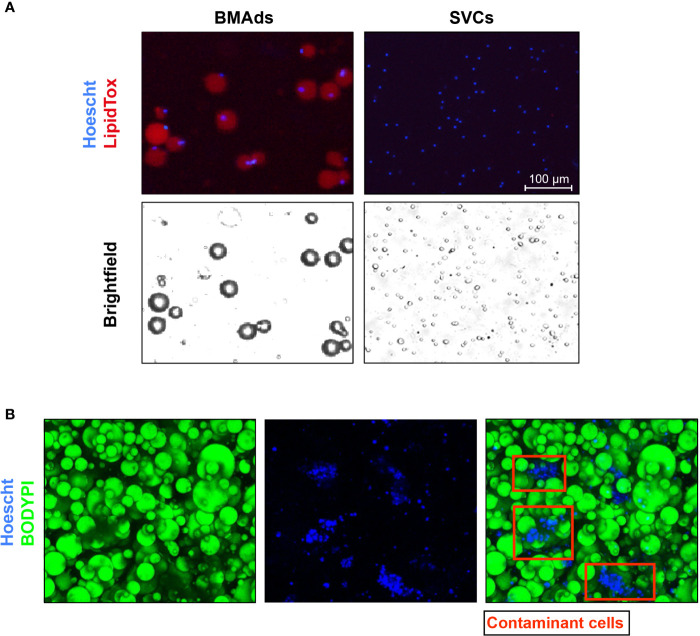
Histological images demonstrating the purity of the BMAds. **(A)** BMAds were stained with Hoechst and LipidTox DeepRed. Stromal-vascular cells (SVCs) were included as a negative control for LipidTox staining. Stained cells were then analysed using a Nexcelom Vision Cellometer. Adipocyte images confirm that all nuclei are associated with unilocular lipid droplets and there are no lipid-free nuclei, suggesting an absence of contaminating non-adipose cell types. Analysis of the SVC fraction confirms that the LipidTox signal is dependent on the presence of lipid droplets. Scale bar represents 100 μm. **(B)** BMAds were stained with BODIPY and Topro 3 to verify by IF the integrity of BMAds (BODIPY staining) and the purity of the adipocyte suspension. Nucleus staining show the presence of contaminant cells attached to adipocytes (red squares).

Immunofluorescence (IF) imaging is suitable to check both the morphology preservation following isolation and the size of BMAds, as well as potential contaminants. For good IF imaging it is better to embed BMAds in a matrix of fibrin gel (53). BMAds should contain a large lipid droplet filled with lipids, which can be stained using a fluorescent dye for neutral lipids such as BODIPY or LipidTox (**Figure 5A**), or the lipid-droplet marker Perilipin 1 (PLIN1) (53, 63). Nuclear staining (using DAPI, Topro 3 or Hoechst) can inform on the presence of contaminant cells that could remain attached to BMAds (**Figure 5B**) (53, 64). Though not yet reported for human BMAds, the presence of specific markers such as CD45 for leukocytes and CD11b for myeloid cells has already been performed for mouse isolated BMAds using immunostaining to discard monocyte or macrophage contaminants (69). Alternatively, flow cytometry analysis following immunolabeling for markers of endothelial cells (CD31), stromal cells (CD105, CD90, CD24) has also been reported to validate the purity of human BMAds (63, 70).

BMAd purity is usually assessed by analyzing the expression of typical transcripts. Adiponectin *(ADIPOQ*), PPARγ (*PPARG*), leptin (*LEP*) (69) and *PLIN1* (63) are used as classical adipocyte markers. Other genes such as integrin alpha-5 (*ITGA5*), *CD11B* (encoded by *ITGAM*) and CD45 (encoded by *PTPRC*) have also been used, respectively, as markers of stromal cells (both for BMSCs and stromal vascular cells within WAT), leukocytes and other hematopoietic cells (51, 65). Based on the specifics of the conducted study, other markers for osteoblasts [e.g. *RUNX2*, Osteocalcin (62)] and for osteoclasts (TRAcP, Cathepsin K) can also be helpful to test cell purity.

##### Evaluation of Cell Viability

Based on previous studies investigating functional assays on BMAds (e.g. lipolysis) (53), metabolic studies require at least 50 µl suspension (around 50,000 cells) per condition and several experimental conditions (with and without pharmacological agents) are needed. However, the number of BMAds at the end of the purification process is generally limited and they are very fragile. Of note, other functional tests with isolated BMAds have already been performed following a 24h culture in various media (62, 63) or ceiling culture (54, 65), yet the impact of this subculture has not been clearly assessed. Therefore, it should be emphasized that these functional metabolic assays are difficult to set up and do not seem appropriate to rapidly check the viability of BMAds. Other developments of functional tests are thus to be pursued based on our growing knowledge of BMAd metabolism.

### 5.2 Isolation and Culture of BMSCs

Within our working group, we reviewed two protocols for the isolation of BMSCs and BMAd-enriched fraction and validation of the purity of BMSCs obtained by BM aspirates from iliac crest or femoral head (55) (**Table 2**). Based on the source of BM aspirate, the yield of isolated BMSCs can differ (BM aspirate under local anesthesia *vs* BM aspirate taken during an orthopedic surgery procedure). In the following paragraphs, we describe a general protocol accompanied with the notes to highlight the critical steps and remarks which need to be considered during isolation.

#### 5.2.1 Isolation and Transports

BMSCs are isolated from BM of “healthy volunteers” (non-relevant diagnosis prior to surgery), from hematological patients at the iliac crest, or from orthopedic patients upon gentle washing of the femoral head (obtained and fragmented into pieces during hip replacement surgery) (**Figures 4B, D**). An aliquot of one ml of BM diluted with heparin can be centrifuged 2,000 g, 10 min at 4°C to preserve BM plasma. After centrifugation, the supernatant (BM plasma) is transferred to a new tube and frozen at -80°C for subsequent analyses.

BM aspirates are obtained by aspiration of 5-10 mL from the iliac crest after infiltration of the area with local anesthetic (lidocaine, 10 mg/mL), in a 20 mL syringe and mixed 1:1 with basal media containing heparin (100 U/mL) or EDTA (1 mM)**^1,2^**.

After the isolation of the BM aspirate, the samples are transferred to a tube prepared with buffer containing anticoagulant and directly processed for isolation of BMSCs and BMAd-enriched fraction. The BM samples should be processed within a couple of hours, otherwise the viability of the cells can be compromised**^3^**.

***Notes*:**

*1- We do not observe a huge difference between heparin or EDTA as a source of anticoagulant in the buffer for transport of BM aspirates regarding the yield and viability of isolated BMSCs. Similar results are obtained by collecting BM aspirates into commercial EDTA tubes or by using 1 mM of EDTA (replacing Heparin) into PBS.*
*2- Before the procedure, Minimum Essential Medium (MEM) is prepared with heparin (100 U/ml) or with 1 mM EDTA and kept at 4°C.*
*3- The BM samples should be ideally transferred at room temperature and processed within two hours. The transport duration and temperature should be recorded, as described previously.*

#### 5.2.2 General Procedure for BMSC and BMAd-Enriched Fraction Isolation

**Reagents and Equipment:**

- Lymphoprep (StemCell, Scintila) (stored at RT, protected from a light) OR Ficoll (Ficoll-Paque™ PLUS (#17-1440-03, GE Healthcare)
- MEM (Invitrogen cat.no. 04193013M, stored at 4°C)
- MEM + 10% Fetal Bovine Serum (FBS) + 1% Penicilin/Streptamycin (stored at 4°C)
- Human Fibroblast Growth Factor (FGF)-2 (R&D system, # 233-FB-010) to add directly to the medium at 1 ng/mL (optional)
- Heparin (1,000 U/ml; stored at 4°C)
- Sterile filter (0.2 μM)
- 10 ml syringe
- Basket filter 100 μM
- Kwill or Falcon plastic tubes
- Small disposable needles
- Pasteur pipette
- Centrifuge with cooling and setting for non-break spinning

**General Protocol (** **Figure 4** **):**

- Dilute harvested BM aspirate 1:1 in PBS (with Ca and Mg), mix gently and immediately divide into two 50 ml Falcon tubes**^4^**.
- Add 10 ml Lymphoprep or 15 ml Ficoll by the kwill plastic tubes, which is placed at the bottom of the tube in order to create a gradient for the separation**^5^**.
- To obtain a floating BMAd-enriched fraction centrifuge samples at 1,050 g for 25-30 minutes at **RT without brake**. For BMSCs, centrifuge samples at 300g 30 min at **RT without brake.** After centrifugation, collect the BMAd-enriched fraction on the top by a pipette **(** **Figures 3B**, **4C**) and follow the steps of washing and harvest in appropriate buffer depending on the subsequent analyses**^6,7^**.
- For the mononuclear cell component containing the BMSC fraction, isolate the buffy coat with a Pasteur pipette and transfer to the prepared 50 ml Falcon tube with preheated MEM medium. The buffy coats from both tubes are combined**^8^**. Higher BMSC yield can be obtained by also recovering the “serum” fraction above the buffy coat and below the floating BMAd-enriched fraction, which contains less cells but relatively enriched in BMSCs.
- Centrifuge tube at 1,050g for BMAds or at 300g for BMSCs for 10 min. Aspirate supernatant, disperse cell pellet in fresh complete medium (~ 20 ml), count the cells and seed them for *in vitro* cultivation^9^.

***Notes:***

*4- Before the gradient separation (by Lymphoprep or Ficoll), the BM sample is filtered with a cell strainer (100 μm) to remove big clumps that can cause disturbances in the gradient. We apply a sterile plunger the filter to break the small aggregates and cell clusters.*
*5- Lymphoprep or Ficoll are aspirated from the bottle with a small disposable needle, which is changed to the kwill plastic needle appropriate for making the gradient.*
*6- BMAd-enriched fraction is washed 2-3 times with PBS/1%BSA and then aliquoted for RNA and protein sample by preservation in Trizol or protein lysis buffer, respectively, and stored at -80°C (more details in* **Table 4** *). Note that this fraction contains a very significant amount of contaminating hematopoietic and immune cells, but it is the only source of enriched BMAd from iliac crest BM aspirates remaining from diagnostic procedures in hematological patients. Results associated to this fraction have to be interpreted within this context.*
*7- The BMAd-enriched fraction can also be preserved for lipid storage. For this purpose, the BMAd-enriched fraction is transferred to an Eppendorf tube, centrifuged at maximum speed for 5 minutes followed by collecting the lipid supernatant into a clean Eppendorf tube, flash freezing by submersion in liquid nitrogen and store at -80°C. Lipid extraction is performed at a subsequent step prior to further analysis (HPLC, lipidomics).*
*8- The serum fraction above the buffy coat can also be collected, as it contains a significant number of BMSCs.*
*9- To improve cell growth, we add hFGF2 to the medium (at 1 ng/ml).*

#### 5.2.3 BMSC Cultivation

BMSCs are cultured at a density 1x10^5^ cells/cm^2^ (1x10^6^ cells per chamber slide)**^10^** in MEM containing 10% fetal bovine serum (FBS, GIBCO) and antibiotics at 1% (penicillin/streptomycin) incubated at 5% CO_2_ and 37°C followed by completely changing the medium once a week**^11^**, while passaging at 70-80% confluence. The cells from passage 0 are cryopreserved in freezing media containing FBS, MEM and DMSO**^12^** (**Table 4**). For cryopreservation, cells in freezing media are immediately transferred into freezer at -80°C for 24 hours before storing them in liquid nitrogen.

Cultured cells are sub-cultured**^13^** and further used for quality control tests (CFU-F, purity assessment, as described below (Section 5.2.5) and in previous literature (8) and subsequently studied in differentiation conditions when appropriate to induce adipogenesis, osteogenesis and/or chondrogenesis. Passage number should be recorded for all experiments**^13^**.

***Notes:***

*10- We usually seed 10 million cells per T75 flask.*
*11- After 4-7 days the stromal cells have attached and started making colonies. Change medium without disturbing or perturbing cells. Media can be switched to Super-MEM (10% FBS/1% pen/strep/glutamax/nonessential amino acids/sodium pyruvate) to enrich the media for essential components needed for BMSC proliferation.*
*12- There are several options to prepare freezing media e.g. 80% FBS, 10% MEM and 10% DMSO (* **Table 4** *) or a variant recipe with 50% MEM, 40% FBS and 10% DMSO.*
*13- The cultured cells from passage 0 are usually sub-cultured 1:2 in order to expand for further analyses and functional assays. Primary BMSCs are easily capable of passaging up to passage 6-7 but with each passage their capacity to proliferate and differentiate into different cell types is decreased, which affects their cellular and molecular properties. Also, they become more senescent as noticed when cells do not double within 4-5 days* (71, 72).

#### 5.2.4 Sample Storage

Storage of BMSCs and BMAd-enriched samples depends on the purpose of the intended analyses (more details in **Table 4**). The stability of RNA or protein samples is superior when the samples are processed completely, rather than storing them in RNA or protein lysis buffer as specified in **Table 4**. The storage of BMSCs is more stable in liquid nitrogen compared to -80°C.

#### 5.2.5 Quality Controls

**Figure 6:**
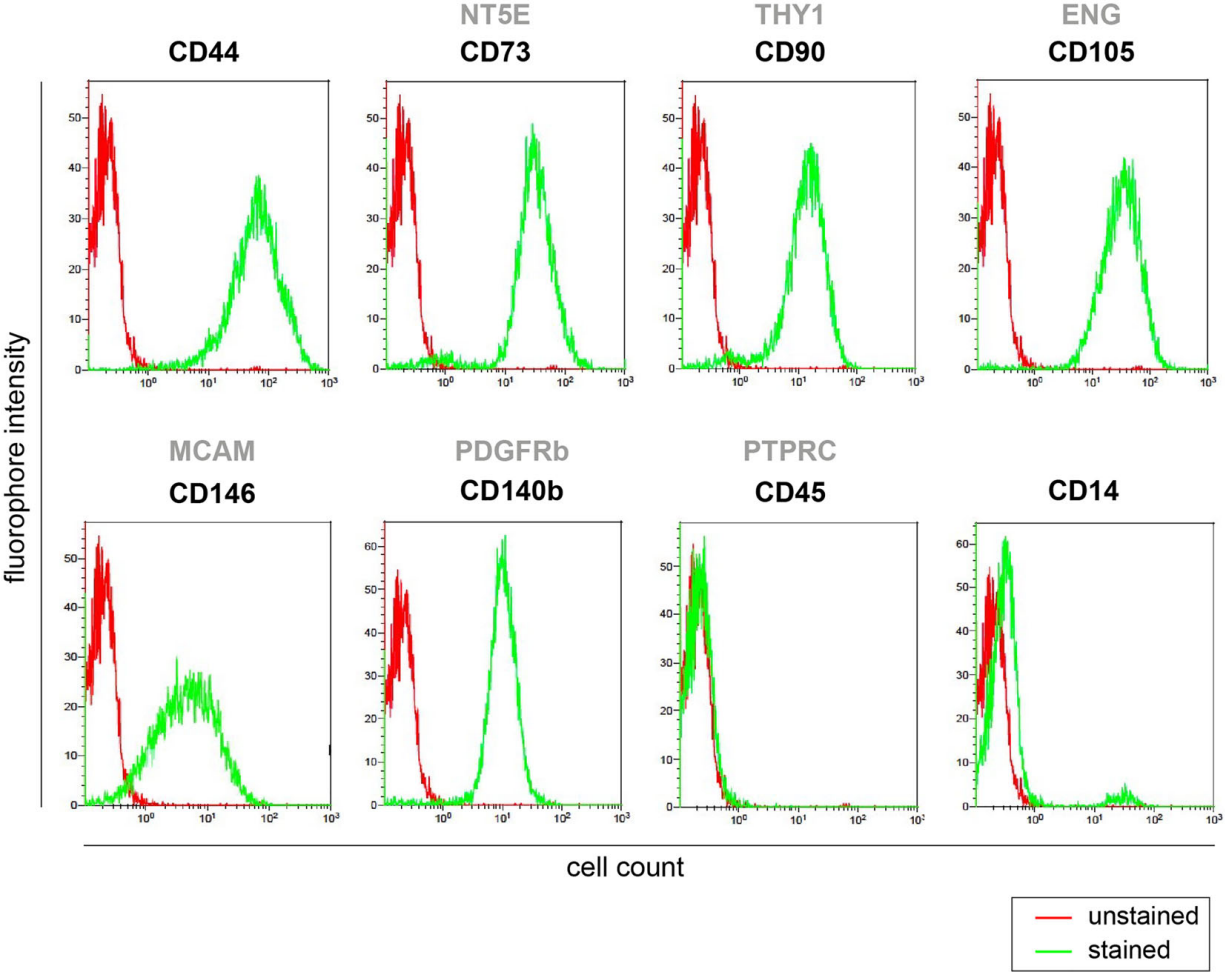
BMSC purity assessment by flow cytometry. Screening of stem cell surface markers expression (positive for CD44, CD73, CD90, CD146, CD140b, CD105, and negative for CD45 and CD14) measured by flow cytometry in primary human BMSCs isolated from iliac crest cultured *in vitro* at passage p0.

**Figure 7:**
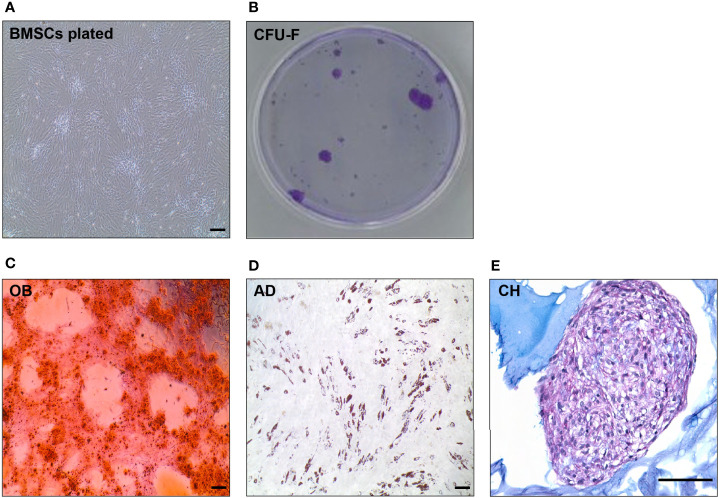
*In vitro* evaluation of BMSC quality (BMSC culture, CFU-C, tri-differentiation). **(A)** Representative picture of spindle-like shape cells in *in vitro* culture of human BMSCs. **(B)** CFU-F of BMSCs after 14 days *in vitro* culture visualized by Violet Blue in a petri dish. **(C)** Alizarin staining of mineralized matrix in 10 days osteoblast (OB) culture differentiated from primary BMSCs. **(D)** Oil Red O staining of neutral lipids in a 10 days adipocyte (AD) culture differentiated from primary BMSCs. **(E)** Alcian Blue staining of a 21 days chondrocyte (CH) culture differentiated from BMSCs. Scale bar represents 200 μm in all the pictures.

There are several methods available to check the quality of isolated BMSC samples [as detailed in (8)]. Representative pictures of validated methods are depicted in **Figures 6**, **7**.

##### Cell Viability

The cell viability of isolated BMSCs can be verified by Trypan blue staining or by flow cytometry using 7-AAD staining (7- amino-actinomycin D; marker of dead cells) or Live/dead viability assay (73).

##### Purity (Hematopoietic, Endothelial Cell Contamination)

The purity of BMSCs can be checked by flow cytometry using CD markers for BMSCs (e.g. CD73, CD271, CD146, CD90, CD105), hematopoietic cells (e.g. CD45 neg), erythroid cells (e.g. CD235a neg) and monocytes/macrophages (e.g. CD14 neg, CD16 neg) (**Figure 6** for representative dot plots for BMSC purity) (56, 59–61).

##### CFU-F Assay

In order to test stem cell potency of isolated BMSCs (**Figure 7A**), we perform a CFU-F assay, i.e. capability of stem/progenitor cells to form colonies. We seed around 1 million isolated BMSC cells in T25 flask and count number of colonies grown after 14 days. CFU-F assays are done through dilution of the test BMSC population and counting at day 14 after fixation and staining with either methylene blue or crystal violet (**Figure 7B**) (56, 59–61).

##### Bi/Tri-Lineage Differentiation Assay

To test differentiation potential of isolated BMSCs (70, 74), we seed and culture the cells in the conditions to differentiate into osteoblast (bone forming cells), adipocyte (BMAT forming cells), chondrocyte (cartilage forming cells) (**Figures 7C–E**). Each of these differentiation protocols can vary among different laboratories (8). For evaluation of osteoblast differentiation, we perform alkaline phosphatase (ALP) activity assay, Alizarin/ALP staining, gene expression of osteoblast genes (e.g. *RUNX*2, Osteocalcin (*BGLAP*), *ALPL*); for adipocyte differentiation these are Oil Red O or Nile Red staining, gene expression of adipocyte genes (*PPARG, LPL, ADIPOQ, LEP*), and for chondrocyte differentiation these are Alcian Blue staining, gene expression of chondrocyte genes (e.g. *SOX9, COL2A1, ACAN*) are evaluated (56, 59–61, 70, 74).

For the purpose of biobanking it is important to keep all necessary information related to the quality of BMSC samples. However, additional comparative studies are still needed to define the crucial parameters that influence BMSC quality, and which therefore are needed for interpreting the analyses of biobank samples.

## 6 BMAS Strategy to Harmonize BMA-Related Biobanking and Future Perspectives

Increased numbers of BMA-related publications in recent years using different sources of BMAT raised important questions about how to compare and reproduce the data in such a young and dynamic field. This raises an opportunity to create BMA biobanks that can provide material for other researchers and generate improved harmonization in future studies. Therefore, a standard protocol would be preferred to allow comparison of samples obtained from different institutions with specifications of critical points, which have been raised in this position paper based on the evaluation of the current literature.

While isolation protocols and collections for BMSCs have been proposed from various laboratories and private companies for long standing, those relative to BMAds are more recent and heterogenous. We currently validated three protocols from which we generated a general procedure to isolate BMAds using collagenase. Yet, it can be acknowledged that this digestion step (i.e. type of collagenase, duration of collagenase treatment, etc.) has to be further standardized and its impact on adipocyte selection, viability and functionality further characterized. When pure BMAd suspension is required or if the BMAds are to be compared with WAT adipocytes, the use of collagenase is recommended to prevent contamination from other cell types and to ensure that differences between adipocyte isolation procedures is not a confounding factor (65).

As technical developments in transcriptomics and other -omics approaches performed in various other tissues could be relevant for future BMAd analyses, we provide the following suggestions regarding next generation sequencing approaches based on preliminary and often unpublished results from different laboratories belonging to the consortium. Indeed, several factors have been determined as critical for single cell RNA sequencing (scRNA-seq) and other -omics approaches. We suggest to minimize as much as possible manipulation of the cell prior to any -omics or transcriptomic approach. A very recent studies suggest that routine steps such as collagenase digestion can have a detrimental effect on the cell’s transcriptome for all single cell analyses and other omics approaches. If to be used, the time of collagenase treatment or other digestion enzymes should be as short as possible as it has been shown to drastically affect the transcriptome, resulting in that the most important gene signature is the digestion itself (75).

Furthermore, a recent study determined that flash freezing (cryopreservation) of cells from BM aspirates prior to scRNA-seq does not affect the transcriptome (76). By doing so, multiple BM samples can be analyzed simultaneously, which would drastically reduce the batch effect that is often visible for single cell RNA-sequencing experiments. Since currently available scRNA-seq analyses are thus far challenging (77), the compatibility of cryopreservation with single nuclei RNA-seq could constitute a very interesting approach to determine and better characterize the BMAT transcriptional landscape. Indeed, it is today impossible to process an entire adipocyte by standard scRNA-seq, since current technologies use pure oil to create the encapsulation droplets. Partial adipocyte lysis, which is hard to avoid due to their natural fragility, prevents the formation of the droplet emulsion and ruins the chip for further use. However, nuclei extraction from BMAds is still very challenging and current procedures still need to be improved. Altogether, the WG suggests to limit the use of collagenase digestion as much as possible while collecting BMAT for these specific transcriptomic analyses since an absolute cell purity is not required and such a digestion step could interfere with subsequent analyses.

Finally, critical differences between our protocols should be further discussed and validated (e.g. precise requirements and specifications for clinical sample isolation and transportation) or improved (easier and standardized assays to assess the quality of samples, increased yield of isolated materials) for harmonization purposes. Meanwhile our current work paves the way for BMAT biobanking perspectives and next generation sequencing. We thus strongly encourage future publications to provide a detailed description of patient characteristics, BMA source and sampling, isolation procedures and validity tests to enhance standardization in the field.

Last but not least, we should pay attention to clinical/molecular data and the anonymization of participants` personal information as they are aimed to be shared among different institutes. At all times, the utmost care should be taken to deidentify the samples obtained from study participants (as discussed in *Section 2.2*). The technological advancements to perform genetic analyses have advanced to such an extent that certain approaches, such as RNA sequencing and single cell sequencing can allow for identification of the individual. Therefore, there is a task at hand to create awareness among researchers to minimize risks of deidentification.

### Future Perspectives

Although we are still several steps away from achieving this, harmonizing protocols globally would greatly enhance the quality and interpretation of BMA-associated outcomes and yield increased impact in the field. A longer-term goal of this WG is to establish a common protocol or minimal conditions that everyone could use. In an attempt to achieve this, one action of the WG will be to setup a study in which the same material will be processed in different labs, using an identical protocol followed by careful evaluation of the outcomes.

It is important to emphasize that at the beginning of creating a BMA biobank one needs to think about preparation of broad consent for future research, which may help to facilitate harmonization as well and, as it is ethically valid, this should be recommended for biobank research (9). In addition, harmonizing sample collection and storage will facilitate medical ethical boards of institutes of collaborating universities about the synchronous sample characteristics of incoming or outgoing samples in case of international studies. One of the other things that the BMAS WG on Biobanking has in mind is to develop a Global Material Transfer Agreement (GMTA) that would allow for easier acceptance and transport of BMA-related samples between institutes across the globe.

Altogether, this position paper should create awareness and facilitate scientists involved in BMA that plan to or continue studies in which collection, storage and analysis of BMA-related samples are involved.

## Author Contributions

SL, MT, BW and BE coordinated the writing and wrote the section on BMAS strategy. FBJ, KI, IP and BE wrote sections on background, informed consent and patient information. TLA with SL and MT wrote the section on biological materials. SL, MT, MR, BW, ON, CA and WC wrote the section on methodology of cell isolation and analysis. FB-J generated the reference library. WC, SL, TA, CA, MT, BW and BE contributed to and composed the figures. All authors contributed to the article and approved the submitted version.

## Funding

MR was funded by the NIH/NIGMS (P20GM121301 and U54GM115516), NIH/NCI (1R37CA245330-01A1), and the American Cancer Society (#133077-RSG-19-037-01-LIB). MT was funded by the Czech Science Foundation (GACR 20-03586S), EFSD/NovoNordisk foundation Future leaders award (NNF20SA0066174). ON and BW were funded by the Swiss National Science Foundation Sinergia program (CRSII5_186271). SL was funded by MSD Avenir Foundation (ADIMETABONE project) and the University of Littoral-Côte d’Opale (ULCO). WC was funded by the Medical Research Council (MR/M021394/1 and MR/S010505/1). CA was funded by La Ligue contre le cancer (Equipe Labellisée) and INCa PLBIO20-028. IP was funded by NIH/NCI (1R01 CA251394 and 1R01 CA181189). KI was funded by the Academy of Finland (325498). TA was funded by the VELUX foundation (25723) and the Region of Southern Denmark (20/14282).

## Conflict of Interest

BE is chair and coordinator of the BMAS Working Group on Biobanking. All authors are members of the BMAS Working Group on Biobanking. BE, WC and ON are members of the BMAS Executive Board. SL, MR and MT are members of the BMAS Scientific Board.

## Publisher’s Note

All claims expressed in this article are solely those of the authors and do not necessarily represent those of their affiliated organizations, or those of the publisher, the editors and the reviewers. Any product that may be evaluated in this article, or claim that may be made by its manufacturer, is not guaranteed or endorsed by the publisher.

## Abbreviations

ADIPOQ, Adiponectin; ADSC, Adipose tissue-Derived Stromal Cell; ALPL, Alkaline Phosphatase; BM, Bone Marrow; BMA, Bone Marrow Adiposity; BMAd, Bone Marrow Adipocyte; BMAS, Bone Marrow Adiposity Society; BMAT, Bone Marrow Adipose Tissue; BMI, Body Mass Index; BMSC, Bone Marrow Stromal Cell; BSA, Bovine Serum Albumin; CFU, Colony Forming Unit; DMEM, Dulbecco’s Modified Eagle’s Medium; FBS, Fetal Bovine Serum; IF, Immunofluorescence; ITGA5, Integrin alpha 5; KRH, Krebs Ringer HEPES; LEP, Leptin; LPL, Lipoprotein Lipase; MEM, Minimum Essential Medium; PBS, Phosphate Buffered Saline; PIF, Patient Information Form; PLIN1, Perilipin 1; PPARG, Peroxisome Proliferator Activated Receptor Gamma; RUNX2, Runt-related transcription factor 2; scRNA-Seq, Single Cell RNA Sequencing; WAT, White Adipose Tissue; WG, Working Group.

## References

[1] Bani Hassan E Ghasem-Zadeh A Imani M Kutaiba N Wright DK Sepehrizadeh T. Bone Marrow Adipose Tissue Quantification by Imaging. Curr Osteoporos Rep (2019) 17(6):416–28. doi: 10.1007/s11914-019-00539-5 31713178

[2] de Paula FJA Rosen CJ. Marrow Adipocytes: Origin, Structure, and Function. Annu Rev Physiol (2020) 82:461–84. doi: 10.1146/annurev-physiol-021119-034513 31702948

[3] Penel G Kerckhofs G Chauveau C. Brief Report From the 4th International Meeting on Bone Marrow Adiposity (Bma2018). Front Endocrinol (Lausanne) (2019) 10:691. doi: 10.3389/fendo.2019.00691 31681168 PMC6813723

[4] Sebo ZL Rendina-Ruedy E Ables GP Lindskog DM Rodeheffer MS Fazeli PK. Bone Marrow Adiposity: Basic and Clinical Implications. Endocr Rev (2019) 40(5):1187–206. doi: 10.1210/er.2018-00138 PMC6686755 31127816

[5] Bartelt A Koehne T Todter K Reimer R Muller B Behler-Janbeck F. Quantification of Bone Fatty Acid Metabolism and Its Regulation by Adipocyte Lipoprotein Lipase. Int J Mol Sci (2017) 18(6):1264. doi: 10.3390/ijms18061264 PMC5486086 28608812

[6] Mate S Kampf M Rodle W Kraus S Proynova R Silander K. Pan-European Data Harmonization for Biobanks in. Appl Clin Inform (2019) 10(4):679–92. doi: 10.1055/s-0039-1695793 PMC6739205 31509880

[7] Bravenboer N Bredella MA Chauveau C Corsi A Douni E Ferris WF. Standardised Nomenclature, Abbreviations, and Units for the Study of Bone Marrow Adiposity: Report of the Nomenclature Working Group of the International Bone Marrow Adiposity Society. Front Endocrinol (Lausanne) (2019) 10:923. doi: 10.3389/fendo.2019.00923 32038486 PMC6993042

[8] Tratwal J Labella R Bravenboer N Kerckhofs G Douni E Scheller EL. Reporting Guidelines, Review of Methodological Standards, and Challenges Toward Harmonization in Bone Marrow Adiposity Research. Report of the Methodologies Working Group of the International Bone Marrow Adiposity Society. Front Endocrinol (2020) 11:65. doi: 10.3389/fendo.2020.00065 PMC7059536 32180758

[9] Hansson MG Dillner J Bartram CR Carlson JA Helgesson G. Should Donors be Allowed to Give Broad Consent to Future Biobank Research? Lancet Oncol (2006) 7(3):266–9. doi: 10.1016/S1470-2045(06)70618-0 16510336

[10] Charmantier I. Carl Linnaeus and the Visual Representation of Nature. Hist Stud Nat Sci (2011) 41(4):365–404. doi: 10.1525/hsns.2011.41.4.365 22363966

[11] Funk VA. Collections-Based Science in the 21st Century. J Syst Evol (2018) 56(3):175–93. doi: 10.1111/jse.12315

[12] Loft S Poulsen HE. Cancer Risk and Oxidative DNA Damage in Man. J Mol Med (Berl) (1996) 74(6):297–312. doi: 10.1007/BF00207507 8862511

[13] Hewitt R Watson P. Defining Biobank. Biopreserv Biobank (2013) 11(5):309–15. doi: 10.1089/bio.2013.0042 24835262

[14] Larsson A. The Need for Research Infrastructures: A Narrative Review of Large-Scale Research Infrastructures in Biobanking. Biopreserv Biobank (2017) 15(4):375–83. doi: 10.1089/bio.2016.0103 28253021

[15] Mayrhofer MT Holub P Wutte A Litton JE. BBMRI-ERIC: The Novel Gateway to Biobanks. From Humans to Humans. Bundesgesundheitsblatt Gesundheitsforschung Gesundheitsschutz (2016) 59(3):379–84. doi: 10.1007/s00103-015-2301-8 26860601

[16] Kinkorova J. Biobanks in the Era of Personalized Medicine: Objectives, Challenges, and Innovation: Overview. EPMA J (2015) 7:4. doi: 10.1186/s13167-016-0053-7 26904153 PMC4762166

[17] Paskal W Paskal AM Debski T Gryziak M Jaworowski J. Aspects of Modern Biobank Activity - Comprehensive Review. Pathol Oncol Res (2018) 24(4):771–85. doi: 10.1007/s12253-018-0418-4 PMC6132819 29728978

[18] Fransson MN Rial-Sebbag E Brochhausen M Litton JE. Toward a Common Language for Biobanking. Eur J Hum Genet (2015) 23(1):22–8. doi: 10.1038/ejhg.2014.45 PMC4266732 24713663

[19] Sudlow C Gallacher J Allen N Beral V Burton P Danesh J. UK Biobank: An Open Access Resource for Identifying the Causes of a Wide Range of Complex Diseases of Middle and Old Age. PloS Med (2015) 12(3):e1001779. doi: 10.1371/journal.pmed.1001779 25826379 PMC4380465

[20] Gottweis H Zatloukal K. Biobank Governance: Trends and Perspectives. Pathobiology (2007) 74(4):206–11. doi: 10.1159/000104446 17709961

[21] Coppola L Cianflone A Grimaldi AM Incoronato M Bevilacqua P Messina F. Biobanking in Health Care: Evolution and Future Directions. J Transl Med (2019) 17(1):172. doi: 10.1186/s12967-019-1922-3 31118074 PMC6532145

[22] Peakman T Elliott P. Current Standards for the Storage of Human Samples in Biobanks. Genome Med (2010) 2(10):72. doi: 10.1186/gm193 20923579 PMC2988449

[23] OECD. OECD Guidelines on Human Biobanks and Genetic Research Databases (2009). Available at: http://www.oecd.org/sti/emerging-tech/44054609.pdf (Accessed 6th of August 2020).

[24] OECD. OECD Best Practice Guidelines for Biological Resource Centres (2007). Available at: http://www.oecd.org/sti/emerging-tech/38777417.pdf (Accessed 6th of August 2020).

[25] Muller H Dagher G Loibner M Stumptner C Kungl P Zatloukal K. Biobanks for Life Sciences and Personalized Medicine: Importance of Standardization, Biosafety, Biosecurity, and Data Management. Curr Opin Biotechnol (2020) 65:45–51. doi: 10.1016/j.copbio.2019.12.004 31896493

[26] Jefford M Moore R. Improvement of Informed Consent and the Quality of Consent Documents. Lancet Oncol (2008) 9(5):485–93. doi: 10.1016/S1470-2045(08)70128-1 18452859

[27] Master Z Nelson E Murdoch B Caulfield T. Biobanks, Consent and Claims of Consensus. Nat Methods (2012) 9(9):885–8. doi: 10.1038/nmeth.2142 22936169

[28] Siminoff LA Wilson-Genderson M Mosavel M Barker L Trgina J Traino HM. Confidentiality in Biobanking Research: A Comparison of Donor and Nondonor Families’ Understanding of Risks. Genet Test Mol Biomark (2017) 21(3):171–7. doi: 10.1089/gtmb.2016.0407 PMC5367914 28121471

[29] Udesky JO Boronow KE Brown P Perovich LJ Brody JG. Perceived Risks, Benefits, and Interest in Participating in Environmental Health Studies That Share Personal Exposure Data: A U.S. Survey of Prospective Participants. J Empir Res Hum Res Ethics (2020) 15(5):425–42. doi: 10.1177/1556264620903595 PMC7429332 32065041

[30] Abbing HD. Unesco. International Declaration on Human Genetic Data. Eur J Health Law (2004) 11(1):93–107. doi: 10.1163/157180904323042399 15285199

[31] WHO. Templates for Informed Consent Forms (2020). Available at: https://www.who.int/groups/research-ethics-review-committee/guidelines-on-submitting-research-proposals-for-ethics-review/templates-for-informed-consent-forms (Accessed 30th of November 2020).

[32] Bleiberg H Decoster G de Gramont A Rougier P Sobrero A Benson A. A Need to Simplify Informed Consent Documents in Cancer Clinical Trials. A Position Paper of the ARCAD Group. Ann Oncol (2017) 28(5):922–30. doi: 10.1093/annonc/mdx050 PMC5406755 28453700

[33] Council of Europe. Guide for the Implementationof the Principle of Prohibition of Financial Gain With Respect to the Human Body and Its Parts From Living or Deceased Donors (2018). Available at: https://rm.coe.int/guide-financial-gain/16807bfc9a (Accessed 06th of May 2021).

[34] Hayflick L. Paying for Tissue: The Case of WI-38. Science (2012) 337(6100):1292. doi: 10.1126/science.337.6100.1292-a 22984048

[35] Truog RD Kesselheim AS Joffe S. Research Ethics. Paying Patients for Their Tissue: The Legacy of Henrietta Lacks. Science (2012) 337(6090):37–8. doi: 10.1126/science.1216888 PMC4256075 22767914

[36] Le Ster C Lasbleiz J Kannengiesser S Guillin R Gambarota G Saint-Jalmes H. A Fast Method for the Quantification of Fat Fraction and Relaxation Times: Comparison of Five Sites of Bone Marrow. Magnet Reson Imaging (2017) 39:157–61. doi: 10.1016/j.mri.2017.03.001 28263827

[37] Costa S Reagan MR. Therapeutic Irradiation: Consequences for Bone and Bone Marrow Adipose Tissue. Front Endocrinol (2019) 10:587. doi: 10.3389/fendo.2019.00587 PMC6727661 31555210

[38] Rharass T Lucas S. Mechanisms in Endocrinology: Bone Marrow Adiposity and Bone, a Bad Romance? Eur J Endocrinol (2018) 179(4):R165–78. doi: 10.1530/EJE-18-0182 30299886

[39] Suchacki KJ Cawthorn WP. Molecular Interaction of Bone Marrow Adipose Tissue With Energy Metabolism. Curr Mol Biol Rep (2018) 4(2):41–9. doi: 10.1007/s40610-018-0096-8 PMC5976678 29888168

[40] Vande Berg BC Gilon R Malghem J Lecouvet F Depresseux G. Correlation Between Baseline Femoral Neck Marrow Status and the Development of Femoral Head Osteonecrosis in Corticosteroid-Treated Patients: A Longitudinal Study by MR Imaging. Eur J Radiol (2006) 58(3):444–9. doi: 10.1016/j.ejrad.2006.01.009 16510260

[41] Veldhuis-Vlug AG Rosen CJ. Clinical Implications of Bone Marrow Adiposity. J Intern Med (2018) 283(2):121–39. doi: 10.1111/joim.12718 PMC5847297 29211319

[42] Lee SH Erber WN Porwit A Tomonaga M Peterson LC. ICSH Guidelines for the Standardization of Bone Marrow Specimens and Reports. Int Jnl Lab Hem (2008) 30:349–64. doi: 10.1111/j.1751-553X.2008.01100.x 18822060

[43] Gillet C Dalla Valle A Gaspard N Spruyt D Vertongen P Lechanteur J. Osteonecrosis of the Femoral Head: Lipotoxicity Exacerbation in MSC and Modifications of the Bone Marrow Fluid. Endocrinology (2017) 158(3):490–502. doi: 10.1210/en.2016-1687 28359085

[44] Miranda M Pino AM Fuenzalida K Rosen CJ Seitz G Rodriguez JP. Characterization of Fatty Acid Composition in Bone Marrow Fluid From Postmenopausal Women: Modification After Hip Fracture. J Cell Biochem (2016) 117(10):2370–6. doi: 10.1002/jcb.25534 PMC4969132 27416518

[45] Pino AM Rios S Astudillo P Fernandez M Figueroa P Seitz G. Concentration of Adipogenic and Proinflammatory Cytokines in the Bone Marrow Supernatant Fluid of Osteoporotic Women. J Bone Miner Res (2010) 25(3):492–8. doi: 10.1359/jbmr.090802 19653807

[46] Kirwan JA Brennan L Broadhurst D Fiehn O Cascante M Dunn WB. Preanalytical Processing and Biobanking Procedures of Biological Samples for Metabolomics Research: A White Paper, Community Perspective (for “Precision Medicine and Pharmacometabolomics Task Group”-The Metabolomics Society Initiative). Clin Chem (2018) 64(8):1158–82. doi: 10.1373/clinchem.2018.287045 29921725

[47] Yin P Peter A Franken H Zhao X Neukamm SS Rosenbaum L. Preanalytical Aspects and Sample Quality Assessment in Metabolomics Studies of Human Blood. Clin Chem (2013) 59(5):833–45. doi: 10.1373/clinchem.2012.199257 23386698

[48] Gonzalez-Dominguez R Gonzalez-Dominguez A Sayago A Fernandez-Recamales A. Recommendations and Best Practices for Standardizing the Pre-Analytical Processing of Blood and Urine Samples in Metabolomics. Metabolites (2020) 10(6):229. doi: 10.3390/metabo10060229 PMC7344701 32503183

[49] Vasikaran S Eastell R Bruyere O Foldes AJ Garnero P Griesmacher A. Markers of Bone Turnover for the Prediction of Fracture Risk and Monitoring of Osteoporosis Treatment: A Need for International Reference Standards. Osteoporos Int (2011) 22(2):391–420. doi: 10.1007/s00198-010-1501-1 21184054

[50] Ferland-McCollough D Masseli D Spinetti G Sambataro M Sullivan N Blom A. MCP-1 Feedback Loop Between Adipocytes and Mesenchymal Stromal Cells Causes Fat Accumulation and Contributes to Hematopoietic Stem Cell Rarefaction in the Bone Marrow of Diabetic Patients. Diabetes (2018) 67(7):1380–94. doi: 10.2337/db18-0044 29703845

[51] Suchacki KJ Tavares AAS Mattiucci D Scheller EL Papanastasiou G Gray C. Bone Marrow Adipose Tissue Is a Unique Adipose Subtype With Distinct Roles in Glucose Homeostasis. Nat Commun (2020) 11(1):3097. doi: 10.1038/s41467-020-16878-2 32555194 PMC7303125

[52] Griffith JF Yeung DKW Ahuja AT Choy CWY Mei WY Lam SSL. A Study of Bone Marrow and Subcutaneous Fatty Acid Composition in Subjects of Varying Bone Mineral Density. Bone (2009) 44(6):1092–6. doi: 10.1016/j.bone.2009.02.022 19268721

[53] Attané C Estève D Chaoui K Iacovoni JS Corre J Moutahir M. Human Bone Marrow Is Comprised of Adipocytes With Specific Lipid Metabolism. Cell Rep (2020) 30(4):949–58.e6. doi: 10.1016/j.celrep.2019.12.089 31995765

[54] Poloni A Maurizi G Serrani F Mancini S Zingaretti MC Frontini A. Molecular and Functional Characterization of Human Bone Marrow Adipocytes. Exp Hematol (2013) 41(6):558–66.e2. doi: 10.1016/j.exphem.2013.02.005 23435314

[55] Tencerova M Frost M Figeac F Nielsen TK Ali D Lauterlein JL. Obesity-Associated Hypermetabolism and Accelerated Senescence of Bone Marrow Stromal Stem Cells Suggest a Potential Mechanism for Bone Fragility. Cell Rep (2019) 27(7):2050–62.e6. doi: 10.1016/j.celrep.2019.04.066 31091445

[56] Caplan AI. Mesenchymal Stem Cells. J Orthop Res (1991) 9(5):641–50. doi: 10.1002/jor.1100090504 1870029

[57] Tanavde V Vaz C Rao MS Vemuri MC Pochampally RR. Research Using Mesenchymal Stem/Stromal Cells: Quality Metric Towards Developing a Reference Material. Cytotherapy (2015) 17(9):1169–77. doi: 10.1016/j.jcyt.2015.07.008 PMC4943322 26276001

[58] Pham TT Ivaska KK Hannukainen JC Virtanen KA Lidell ME Enerback S. Human Bone Marrow Adipose Tissue Is a Metabolically Active and Insulin-Sensitive Distinct Fat Depot. J Clin Endocrinol Metab (2020) 105(7):2300–10. doi: 10.1210/clinem/dgaa216 PMC7247553 32311037

[59] Bianco P Gehron Robey P. Marrow Stromal Stem Cells. J Clin Invest (2000) 105(12):1663–8. doi: 10.1172/JCI10413 PMC378520 10862779

[60] Bianco P Riminucci M Gronthos S Robey PG. Bone Marrow Stromal Stem Cells: Nature, Biology, and Potential Applications. Stem Cells (Dayton Ohio) (2001) 19(3):180–92. doi: 10.1634/stemcells.19-3-180 11359943

[61] Bianco P Robey PG Simmons PJ. Mesenchymal Stem Cells: Revisiting History, Concepts, and Assays. Cell Stem Cell (2008) 2(4):313–9. doi: 10.1016/j.stem.2008.03.002 PMC2613570 18397751

[62] Goto H Hozumi A Osaki M Fukushima T Sakamoto K Yonekura A. Primary Human Bone Marrow Adipocytes Support TNF-α-Induced Osteoclast Differentiation and Function Through RANKL Expression. Cytokine (2011) 56(3):662–8. doi: 10.1016/j.cyto.2011.09.005 21963155

[63] Miggitsch C Meryk A Naismith E Pangrazzi L Ejaz A Jenewein B. Human Bone Marrow Adipocytes Display Distinct Immune Regulatory Properties. EBioMedicine (2019) 46:387–98. doi: 10.1016/j.ebiom.2019.07.023 PMC6711052 31327694

[64] Attané C Estève D Moutahir M Reina N Muller C. A Protocol for Human Bone Marrow Adipocyte Isolation and Purification. Star Protoc (2021) 2(3). doi: 10.1016/j.xpro.2021.100629 PMC8246640 34235494

[65] Mattiucci D Maurizi G Izzi V Cenci L Ciarlantini M Mancini S. Bone Marrow Adipocytes Support Hematopoietic Stem Cell Survival. J Cell Physiol (2018) 233(2):1500–11. doi: 10.1002/jcp.26037 28574591

[66] Rodbell M. Localization of Lipoprotein Lipase in Fat Cells of Rat Adipose Tissue. J Biol Chem (1964) 239:753–5. 14154450

[67] Craft CS Scheller EL. Evolution of the Marrow Adipose Tissue Microenvironment. Calcif Tissue Int (2016) 100:461–75. doi: 10.1007/s00223-016-0168-9 PMC5618436 27364342

[68] Lafontan M. Historical Perspectives in Fat Cell Biology: The Fat Cell as a Model for the Investigation of Hormonal and Metabolic Pathways. Am J Physiol Cell Physiol (2012) 302(2):C327–59. doi: 10.1152/ajpcell.00168.2011 21900692

[69] Liu L-F Shen W-J Ueno M Patel S Kraemer FB. Characterization of Age-Related Gene Expression Profiling in Bone Marrow and Epididymal Adipocytes. BMC Genomics (2011) 12:212. doi: 10.1186/1471-2164-12-212 21545734 PMC3113784

[70] Dominici M Le Blanc K Mueller I Slaper-Cortenbach I Marini F Krause D. Minimal Criteria for Defining Multipotent Mesenchymal Stromal Cells. The International Society for Cellular Therapy Position Statement. Cytotherapy (2006) 8(4):315–7. doi: 10.1080/14653240600855905 16923606

[71] Andrzejewska A Catar R Schoon J Qazi HT Sass FA Jacobi D. Multi-Parameter Analysis of Biobanked Human Bone Marrow Stromal Cells Shows Little Influence for Donor Age and Mild Comorbidities on Phenotypic and Functional Properties. Front Immunol (2019) 10:2474. doi: 10.3389/fimmu.2019.02474 31781089 PMC6857652

[72] Vidal MA Walker NJ Napoli E Borjesson DL. Evaluation of Senescence in Mesenchymal Stem Cells Isolated From Equine Bone Marrow, Adipose Tissue, and Umbilical Cord Tissue. Stem Cells Dev (2012) 21(2):273–83. doi: 10.1089/scd.2010.0589 21410356

[73] Xiao M Dooley DC. Assessment of Cell Viability and Apoptosis in Human Umbilical Cord Blood Following Storage. J Hematother Stem Cell Res (2003) 12(1):115–22. doi: 10.1089/152581603321210190 12662442

[74] Viswanathan S Shi Y Galipeau J Krampera M Leblanc K Martin I. Mesenchymal Stem *Versus* Stromal Cells: International Society for Cell & Gene Therapy (ISCT^®^) Mesenchymal Stromal Cell Committee Position Statement on Nomenclature. Cytotherapy (2019) 21(10):1019–24. doi: 10.1016/j.jcyt.2019.08.002 31526643

[75] Machado L Geara P Camps J Dos Santos M Teixeira-Clerc F Van Herck J. Tissue Damage Induces a Conserved Stress Response That Initiates Quiescent Muscle Stem Cell Activation. Cell Stem Cell (2021) 28(6):1125–35. doi: 10.1016/j.stem.2021.01.017 33609440

[76] Chen D Abu Zaid MI Reiter JL Czader M Wang L McGuire P. Cryopreservation Preserves Cell-Type Composition and Gene Expression Profiles in Bone Marrow Aspirates From Multiple Myeloma Patients. Front Genet (2021) 12:663487. doi: 10.3389/fgene.2021.663487 33968139 PMC8099152

[77] Deutsch A Feng D Pessin JE Shinoda K. The Impact of Single-Cell Genomics on Adipose Tissue Research. Int J Mol Sci (2020) 21(13):4773. doi: 10.3390/ijms21134773 PMC7369959 32635651

